# The Hunger Games: Stable Isotopes Indicate Winter Inter‐Guild Competition for Resources by Marine Meso‐Predators in the Sub‐Arctic North Pacific

**DOI:** 10.1002/ece3.70535

**Published:** 2024-11-26

**Authors:** Genyffer C. Troina, Evgeny A. Pakhomov, Laurie Weitkamp, Aleksey Somov, Brian P. V. Hunt

**Affiliations:** ^1^ Institute for the Oceans and Fisheries University of British Columbia (UBC) Vancouver British Columbia Canada; ^2^ Department of Earth, Ocean and Atmospheric Sciences University of British Columbia Vancouver British Columbia Canada; ^3^ Haikai Institute Heriot Bay British Columbia Canada; ^4^ Northwest Fisheries Science Center, National Marine Fisheries Service National Oceanic and Atmospheric Administration Newport Oregon USA; ^5^ Pacific Scientific Research Fisheries Center (TINRO‐Center) Vladivostok Russia; ^6^ School of Fisheries and Ocean Sciences University of Alaska Fairbanks Juneau Alaska USA

**Keywords:** fish, *Oncorhynchus* spp., open ocean, species interactions, squid, trophic ecology

## Abstract

Interspecific competition can significantly impact marine ecosystems by affecting species distributions and abundances. Understanding how sympatric species utilize available food helps identify potential competition and its effects when resources are limited. Here, we applied a suite of analytical methods (diet analysis, stable isotopes, and biomass estimates) to identify potential competitive interactions among North Pacific pelagic predators. Samples were collected in the Gulf of Alaska during the winter of 2019. Environmental conditions and food web structure (prey consumption, species biomass, and isotopic niche overlap) varied across the region. Several squid and myctophid species occupied similar trophic positions and had high isotopic nice overlap with Pacific salmon. The intensity of these interactions differed between the northwest and southeast Gulf of Alaska. For example, there was a substantial isotopic niche overlap between sockeye salmon and the squid *Onychyoteuthis borealijaponica* in the southeast, while chum salmon exhibited considerable niche overlap with various species in both areas. Our results demonstrate that, as the biomass of non‐salmonid competitors may exceed that of Pacific salmon, these interactions must be considered when assessing salmon production on the high seas. Regional differences in trophic interactions demonstrate that the open ocean northeast Pacific is more dynamic than previously proposed, and knowledge of salmon rearing locations could improve production estimates. Further research on regional ocean properties and their effects on trophic ecology is needed to understand how salmon will respond to climate‐driven changes in ocean conditions. This study provides the first analysis of pelagic food webs in the North Pacific high seas during winter, highlighting significant intra‐guild competition among meso‐predators. The effects of this competition on production are difficult to assess using empirical approaches due to the inaccessibility of the region. We propose the application of the trophic interactions identified here to explore these effects using food web models.

## Graphical abstract

There is high niche overlap and potential for competition between Pacific salmon and non‐salmonid meso‐predators in the open ocean during winter. Food web structure and the level of niche overlap varied spatially and depending on the species. The biomass of the competitor species may exceed that of Pacific salmon, with potential implications for Pacific salmon production.

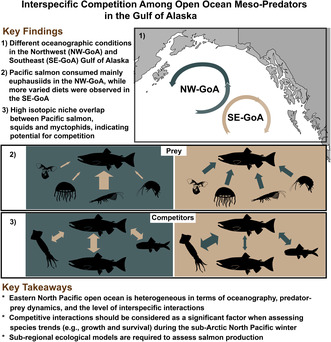

## 1 Introduction

Competitive interactions play a fundamental role in shaping populations, communities, and corresponding food webs. This can occur through density‐dependent processes among coexisting species that require the same resources. The potential for resource competition is high, for example, when species exhibit significant overlap in the food they eat, such as the type or size of consumed prey and when resources are limited. In some cases, inter‐guild competition between coexisting species may help to keep the abundance of the different competitor species in check (Brown and Davidson 1977). In other cases, however, such interactions may result in winners and losers, in which competition has a positive and negative effect, respectively, on a species' ability to meet its nutritional requirements and reproduce, ultimately affecting its recruitment success (Ruggerone and Connors 2015). The level of competition may change seasonally (Andersen et al. 2007) and interannually (Ruggerone et al. 2003), depending on competing species' life‐cycles and trends in environmental conditions that drive prey abundance and species movement patterns. Describing the feeding behavior and resource utilization by sympatric species is essential for recognizing interspecific competition and its potential effects, especially when resources become scarce, or competitor population's densities change due to shifts in species distributions (Kordas, Harley, and O'Connor 2011). Under current anthropogenic‐related environmental changes, there is a pressing need to understand the role of competitive interactions in mediating population and food web responses in the open ocean environments, a research area that remains under explored.

The open ocean waters in the Gulf of Alaska represent an important habitat for numerous commercially important fishes, including Pacific salmon (*Oncorhynchus* spp.) (Beamish et al. 1999; Orlov and Tokranov 2019; Shuntov and Temnykh 2008). Many species of Pacific salmon spend several years in high‐seas habitat during the marine phase of their life cycle, where they grow and mature before returning to their natal rivers to spawn (Beamish 2018; Groot and Margolis 1991; Quinn and Dittman 1990). Salmon are meso‐predators, occupying intermediate trophic levels in the pelagic food webs. Numerous studies utilizing gut content and stable isotope analyses have suggested competition for food among Pacific salmon species (Graham, Pakhomov, and Hunt 2021; Johnson and Schindler 2009; Kaeriyama et al. 2000; Qin and Kaeriyama 2016; Tadokoro et al. 1996; Welch and Parsons 1993). Specifically, these studies have pointed to resource competition between chum (*Oncorhynchus keta*), pink (*Oncorhynchus gorbuscha*), and sockeye (*Oncorhynchus nerka*) salmon. Nevertheless, the severity of inter‐species competition in different parts of the North Pacific Ocean is under debate (Northern Hemisphere Pink Salmon Expert Group 2023). While Russian studies suggest that the Pacific northwest (compared to the northeast) provides a more stable environment for Pacific Salmon that decreases both inter‐ and intraspecific competition (Kuznetsova 2015; Naydenko and Khoruzhiy 2014; Naydenko and Temnykh 2016; Naydenko and Somov 2022; Shuntov, Temnykh, and Ivanov 2017; Shuntov and Ivanov 2019; Volkov 2014; Zavolokin, Radchenko, and Kulik 2014), these interactions remain largely unassessed in the less productive north‐eastern Pacific Ocean.

The high abundance of micro‐nektonic (2–20 cm in size) cephalopods and myctophid species in the sub‐Arctic north‐eastern Pacific raises important questions about the extent of interspecific competition affecting salmon growth. Numerous species of micronekton, including squids and myctophids, have been documented as part of the diet of Pacific salmon (Graham, Pakhomov, and Hunt 2021; Kaeriyama et al. 2004, 2000; Qin and Kaeriyama 2016; Volkov 2014, 2016). However, the salmon diet includes a substantial fraction of lower trophic level zooplankton prey which are also preyed on by micronekton (Chuchukalo 2006; Volkov 2014, 2016). The role of micronekton species may therefore shift from prey to competitors as they grow, potentially resulting in significant competition within these ecosystems. Currently, however, our understanding of potential interactions among and between meso‐predator species is hindered by the limited knowledge about the feeding habits of meso‐predator species in the North Pacific open waters (*Micronekton of the North Pacific*, 2005). This is particularly true for the winter period when many young salmonids experience the open ocean for the first time (Myers et al. 2016).

The Gulf of Alaska experiences strong seasonality in oceanic conditions, with reduced productivity due to strong vertical mixing and reduced day length during winter (Childers, Whitledge, and Stockwell 2005), and peaks in plankton biomass during spring and summer (Coyle and Pinchuk 2003). While past research has examined the feeding ecology of Pacific salmon, most of this work involved samples collected during the warmer seasons (spring and summer) (Kaeriyama et al. 2000; Tadokoro et al. 1996; Welch and Parsons 1993), with little focus on the interactions between Pacific salmon and other non‐salmonid meso‐predators (*Micronekton of the North Pacific*, 2005). Competition for resources may be particularly important during food limited winter months, with potential implications for species' growth and survival. Therefore, it is important to identify potential competitors and their role in shaping species distribution and abundance during this critical period.

In addition to seasonal variability, recent research has shown spatial heterogeneity in the ocean conditions in the Gulf of Alaska that could have implications for food web properties. The *International Year of the Salmon* (IYS) expedition conducted a mesoscale survey in the Gulf of Alaska in the winter of 2019, which found that the region can be broadly separated into northwest and southeast areas (Pakhomov et al. 2019). The northwest area had relatively lower surface water temperatures, higher salinity, and higher nutrient concentrations compared to the southeastern Gulf of Alaska. These oceanographic differences are likely linked to variations in the food web base, as the biomass and distribution of major zooplankton groups also varied spatially (Pakhomov et al. 2019). Such gradients in oceanographic conditions and baseline organisms need to be taken into account when assessing food web dynamics and trophic interactions. Understanding current levels of interspecific trophic interactions and how they vary along gradients of ocean conditions may provide foundational knowledge to better assess the potential implications of future climate‐related changes on the trophic dynamics of these ecosystems.

Investigating the winter ecology of marine predators in the high seas can be particularly challenging due to the harsh weather conditions and distance from shore, posing significant difficulties in carrying out scientific surveys. The 2019 IYS expedition provided a unique opportunity to obtain samples of pelagic organisms and assess the conditions they experience during winter months in the North Pacific Ocean (Pakhomov et al. 2019). In this study we analyzed samples collected during this expedition with the objectives to (1) describe the trophic ecology of North Pacific meso‐predators and (2) identify the potential competitors of Pacific salmon during this critical period in the high seas. To achieve these objectives, we analyzed carbon (ẟ^13^C) and nitrogen (ẟ^15^N) stable isotopes in different meso‐predator species to (1) estimate their trophic positions and assess isotopic similarity (i.e., isotopic niche overlap) of key species and (2) identify salmon competitors in the high seas (i.e., species with similar trophic positions and high isotopic niche overlap). Additionally, we investigated these species' biomass distribution patterns to assess whether isotopically similar species were using resources in the same areas or segregating spatially to minimize potential competition. We expected to see (1) spatial variability in species' trophic position, given the differences in ocean conditions and zooplankton communities within the Gulf of Alaska; (2) a high isotopic niche overlap among pink, sockeye, and chum salmon; and (3) substantial isotopic niche overlap between these three Pacific salmon species and the micronekton as they feed on limited resources available during winter. Identifying the level of potentially competitive interactions among North Pacific meso‐predators will contribute to structuring food web models and can be applied to investigate community responses to climate change.

## 2 Materials and Methods

### 2.1 Survey Area, Sample Collection, and Processing

**FIGURE 1:**
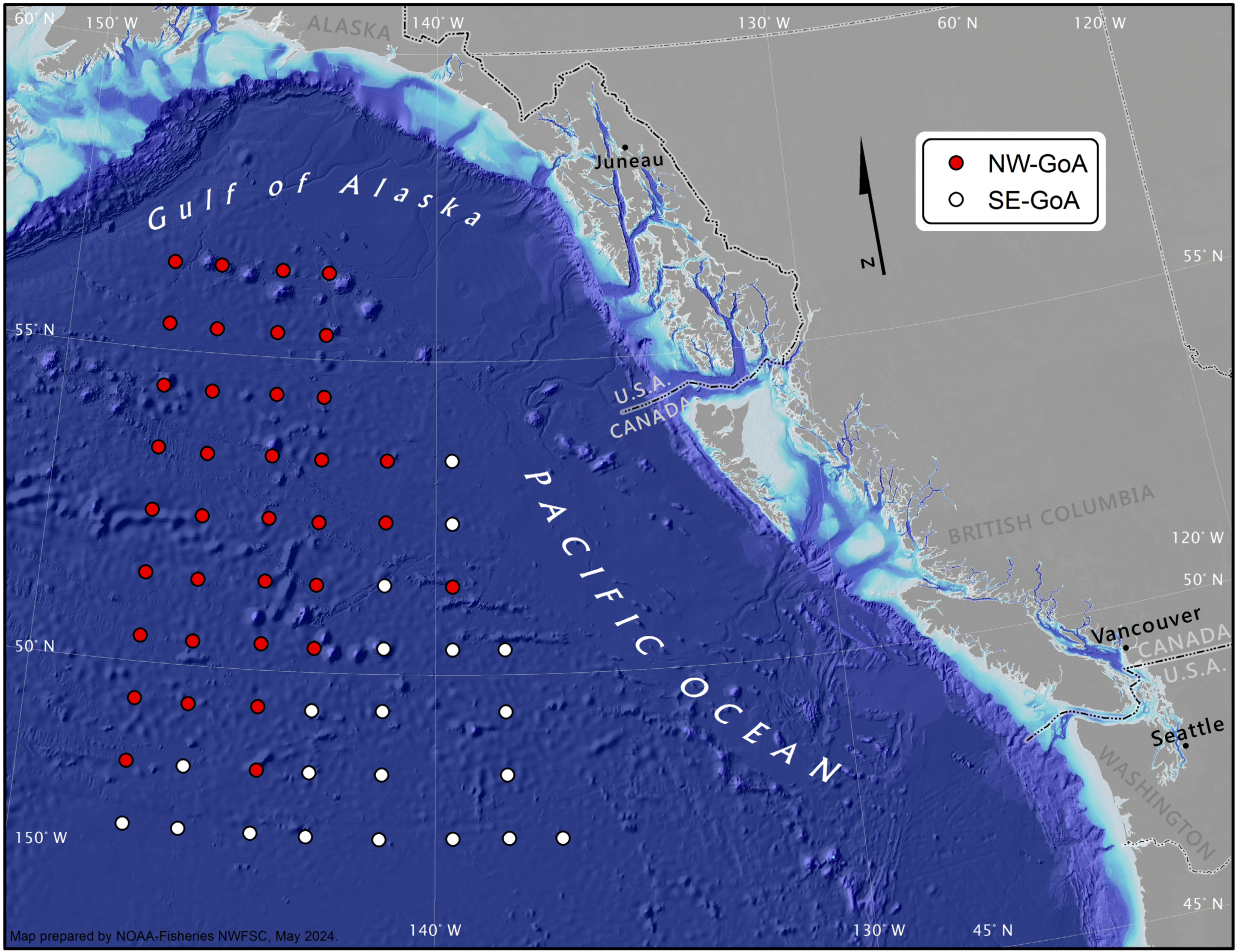
Oceanographic stations sampled in the Northwest (NW‐GoA) and Southeast (SE‐GoA) Gulf of Alaska during the International Year of the Salmon expedition to the high seas in the winter of 2019.

An area of approximately 697,500 km^2^ of the Gulf of Alaska (Figure 1) was surveyed during February and March 2019, onboard of the chartered R/V *Professor Kaganovsky*. A total of 58 stations were sampled between 56° N and 47° N, and 147° W and 136° W.

#### 2.1.1 Water Chemistry

Water samples and data on temperature and salinity were obtained by deploying a Niskin rosette equipped with a SeaBird CTD 911 plus (conductivity, temperature, and depth) to 1000 m depth at every station. Water samples were collected at the surface (~5), 25, 50, 75, 100, 150, 200, 400, 600, and 1000 m depths to measure nutrient concentrations (silicate, Si; dissolved inorganic phosphate, DIP; dissolved inorganic nitrogen, DIN; nitrite ${\mathrm{NO}}_{2}^{-}$; and nitrate, ${\mathrm{NO}}_{3}^{-}$).

#### 2.1.2 Zooplankton

*Z*ooplankton samples were collected with a Bongo net (50 cm mouth diameter and 236 μm mesh) hauled vertically from 250 m depth. The zooplankton from one side of the Bongo net sample at each station was size fractionated using 4, 2, 1, 0.5, and 0.25 mm mesh size, and then frozen at −40°C.

#### 2.1.3 Meso‐Predators

Samples of salmon, squids, jellyfish, myctophids, and other fishes were obtained using a rope trawl (RT 80/396) (~120 m^2^, 30 m deep × 40 m wide) deployed at each station and towed at the surface at 4–5 knots for 1 h. All organisms caught in the trawl were identified to the lowest practical taxon (i.e., species, except for some invertebrates), enumerated, and measured (length [total, fork, mantle, or bell diameter as appropriate] to the nearest 1 mm, and weight to the nearest 1 g). For salmonids, a 2 × 2 cm piece of muscle tissue was collected from above the lateral line and in front of the dorsal fin and stored at −40°C. Salmon stomachs were collected and analyzed onboard using express methods developed in Russian surveys (Volkov 2008), to assess diet through the identification of prey remains. This technique involves stomach grouping by species in 10 cm size classes (10–25 stomachs by group) with further prey identification, weighting, and averaging. For large non‐salmonid fishes, a muscle sample was collected in the same way as for salmonids samples. A 2 × 2 cm piece of muscle from the anterior dorsal margin of the mantle was collected from squids, and jellyfish were collected either whole or piece in the case of large specimens. For micronekton species, either a piece of muscle was sampled (e.g., myctophids posterior region and squids' mantle) or the specimen was analyzed whole (e.g., krill and small jellyfish). All samples were stored frozen at −20°C.

### 2.2 Stable Isotope Analysis and Data Treatment

Tissue samples were either oven‐dried at 50°C (zooplankton, squids, myctophids, and non‐salmonid fishes) or freeze‐dried (salmon and jellyfish), and then homogenized to a fine powder using mortar and pestle. Approximately 1 mg of each sample was encapsulated in tin caps and sent for carbon and nitrogen analysis at the University of California, Davis stable isotope facility (Davis, CA, USA). Tissue samples were analyzed using an elemental analyzer (PDZ Europa ANCA‐GSL) interfaced with an isotope ratio mass spectrometer (PDZ Europa 20‐20, Sercon Ltd., Cheshire, UK). Samples were analyzed interspersed with multiple replicates of laboratory reference materials that have been calibrated against international reference materials. The mean standard deviation for the internal reference materials was ≤ ±0.08‰ for ẟ^13^C and ≤ ±0.07‰ for ẟ^15^N. Reported isotope data are expressed in the δ notation relative to the international standards Vienna Pee Dee Belemnite (VPDB) for carbon and Air for nitrogen. Mathematical lipid correction was applied to the ẟ^13^C values of fish samples that had C:N > 3.0 (Kiljunen et al. 2006). No lipid corrections were applied to squid or jellyfish samples because they have low lipid content (Kariotoglou and Mastronicolis 2001; Lucas 2008).

### 2.3 Data Analysis

#### 2.3.1 Environmental Data

Hierarchical cluster analysis was used to test spatial groupings of stations based on environmental parameters. We used data on surface water (5 m) temperature, salinity, and nutrient (Si, DIP, DIN, ${\mathrm{NO}}_{2}^{-}$, and ${\mathrm{NO}}_{3}^{-}$) concentrations. Data were first normalized using the *scale()* function in R, then the Euclidean distance was calculated between each pair of oceanographic stations using the *dist()* function. We then used the *hclust()* function (“complete” method) to perform a hierarchical cluster analysis based on the distance matrix. This analysis separated stations into two broad clusters, described further in the results section, located either in the northwest Gulf of Alaska (NW‐GoA) or in the southeast Gulf of Alaska (SE‐GoA) (Figure 1). Subsequently, food web isotope data were analyzed separately for each region.

#### 2.3.2 Salmon Diet

Stomach content data were obtained for the five Pacific salmon species analyzed here and provided qualitative (e.g., prey identified to the species level) and quantitative (percentage weight of prey consumed) dietary information. We applied bipartite network analysis (predator–prey‐webs) to identify the trophic links among the different salmon species in the NW‐GoA and SE‐GoA based on the proportion of prey found in the gut of each species. The analysis was completed using the package bipartite (Dormann et al. 2008).

#### 2.3.3 Salmon Size Classes

We tested the relationship between ẟ^13^C and ẟ^15^N values and length (1) to ensure that data were representative of the isotopic values of diet assimilated in the open ocean, especially for the first‐year Pacific salmon and (2) to account for size‐differences in trophic interactions among salmon and non‐salmon species. The smaller chum salmon (*N* = 13, length ≤ 300 mm) had higher ẟ^13^C and ẟ^15^N values than the larger specimens, indicating that their isotopic values were influenced by the period spent in coastal and shelf waters prior to migrating to the open ocean. Therefore, these specimens were removed from analyses. Sockeye salmon sampled in the NW‐GoA were divided into two size classes: specimens with lengths between 300 and 400 mm, and those larger than 400 mm. This subdivision was not possible in the SE‐GoA due to the small number of samples (*N* = 5). Correlations between ẟ^13^C and ẟ^15^N values with length were not significant for pink, coho, or chum (after removal of the small specimens) salmon, thus these species were analyzed without distinction between size classes.

#### 2.3.4 Trophic Position

We used the ẟ^15^N values of 0.25 mm zooplankton size‐fraction as the baseline to estimate the trophic positions (TrP) of organisms sampled in the Gulf of Alaska. This was done using a fixed trophic discrimination factor (TDF) of 3.4‰, applied to the equation proposed by Post (2002): TrP = ([ẟ^15^N_consumer_ − ẟ^15^N_baseline_] + TrP_baseline_)/TDF, where ẟ^15^N_baseline_ and TrP_baseline_ represent, respectively, the nitrogen isotope values and trophic position of the zooplankton 0.25 mm size‐fraction, assumed to have a TrP = 2. As baseline ẟ^15^N values differed between the NW‐GoA and SE‐GoA regions, we used the mean ẟ^15^N values for the 0.25 mm zooplankton size‐fraction sampled within each area to estimate the TrP of organisms, considering the areas they were caught.

We also applied a Bayesian framework to estimate trophic position based on species' carbon and nitrogen stable isotopes using the one‐source model in the R package *tRophicPosition* (Quezada‐Romegialli et al. 2018), including only species with sample size *N* > 3. In the Bayesian model, we used the default TDF of 0.4‰ (SD 1.3) for ẟ^13^C and 3.4‰ (SD 0.98) for ẟ^15^N (Post 2002). This approach estimates posterior distributions of trophic positions with credibility intervals, allowing for statistical comparisons between paired species. The model was run with 500,000 iterations, 5 chains, 20,000 adaptive samplings, and 100,000 burn‐in. We used the function *pairwiseComparisons* to statistically compare the posterior distributions of the estimated trophic positions between every pair of species. We assessed the probability that one species had a trophic position higher than or equal to the TrP in a different species for all pairwise comparisons. Additionally, we used the Bhattacharyya coefficient to calculate the probability of overlap between the posterior distributions of paired species. Unfortunately, the small sample size obtained for Chinook salmon did not allow us to estimate these statistics for this species. For this species, TrP estimates are based on the model by Post (2002), which allows for TrP to be calculated using a sample size as low as 1. Lastly, a hierarchical cluster analysis was applied to assess the level of similarity in the trophic positions of the different species within the NW‐GoA and SE‐GoA Gulf of Alaska food webs. Species identified as having similar trophic positions to salmon were then used in SIBER models (see below) to further assess the level of competition among them.

#### 2.3.5 Isotopic Similarity and Niche Overlap

The isotopic niche space and overlap among species were investigated using the SIBER (Stable Isotope Bayesian Ellipses in R, Jackson et al. 2011) package in R, including only species with similar trophic positions to those of the five salmon species, as identified by the hierarchical cluster analysis based on estimated trophic positions. Due to the small sample size (*N* < 3) per region for some species of jellyfish and squids, individuals were grouped to have enough samples to run SIBER models: hydromedusae included *Aequorea* sp., and *Calycopsis simulans* in both NW‐GoA and SE‐GoA; and the squids *Abraliopsis felis* and *Taonius borealis* were grouped together in the SE‐GoA (Squids Group). A few species could not be analyzed in the SIBER models due to the small sample size and inability to group in ecological meaningful groups. That included the cartilaginous fish *Squalus acanthias* (*N* = 2), and the squids *Gonatus madokai* (*N* = 1), *Onykia robusta* (*N* = 1), and *Watasenia scintillans* (*N* = 2). The isotopic niche area was estimated based on the standard ellipses that represent the area in the isospace encompassing 40% of the bivariate (ẟ^13^C and ẟ^15^N) data. We present the standard ellipse area corrected for small sample size (SEA_C_) and the Bayesian standard ellipse area (SEA_B_). The isotopic niche overlap of paired species was calculated between the corresponding 95% prediction ellipses, using the *maxLikOverlap* function. We report the overlap as the proportion of the non‐overlapping area between every pair of ellipses.

#### 2.3.6 Biomass Distribution

We used biomass data to explore whether species with significant isotopic niche overlap also shared spatial habitat or were segregated spatially. The methodology for estimating biomass is outlined in Pakhomov et al. (2019). Briefly, we calculated the biomass (t/km^2^) of organisms by multiplying catch values by the area swept (km^2^) and catchability coefficient (Pakhomov et al. 2019). Each trawl station was assigned to its Voronoi polygon (see figure 16 in Pakhomov et al. 2019). The total biomass for a given species within the Voronoi polygon was determined by multiplying its biomass at each station by the assigned Voronoi polygon area (km^2^). To calculate the total biomass within the entire study area, we aggregated the total biomass of each selected Voronoi polygon. Due to diel vertical migration, several species were exclusively captured at night trawls. Therefore, to estimate species' total biomass in each area, we used only the night trawl data to estimate biomass per km^2^, which was then extrapolated to encompass the entire surveyed area in each region. We used the biomass data of each species to assess the correlation between paired species over the entire study area (i.e., no distinction between NW‐GoA and SE‐GoA). We present the correlation matrix calculated using the data from the night trawls, but correlations using the entire dataset (day and night trawls) are shown in Appendix S1. We used the function *rcorr()* in the R package *Hmisc* (Harrell Jr. 2023) to calculate the correlation matrix for all possible pairs of species. This function returns the correlation coefficients and the significance levels. Finally, we present the results as the total biomass of each species per region in tons per unit area (km^2^) of the Voronoi polygon surveyed in the night trawls, as well as the total biomass estimated for the entire surveyed area of Voronoi polygon (day and night stations) in each region. This is because the total area of the Voronoi polygon surveyed was different between the NW‐GoA (408,445 km^2^) and SE‐GoA (292,166 km^2^).

## 3 Results

### 3.1 Environmental Data

Two oceanographically distinct areas were identified based on the environmental data collected at each oceanographic station, demonstrating a marked northwest to southeast pattern (Figure 1). The stations from the NW‐GoA had relatively lower temperatures, higher salinity, and higher nutrient concentrations than those in the SE‐GoA (Table 1).

**TABLE 1:** Environmental conditions were measured in the winter of 2019 during the International Year of the Salmon expedition to the Gulf of Alaska.

Region	NW‐GoA		SE‐GoA		*p*
	Mean	SD	Mean	SD	
Temperature (°C)	6.1	0.5	7.5	0.5	**< 0.001**
Salinity (psu)	32.5	0.1	32.4	0	**< 0.001**
Si (μM/L)	21.6	2.9	13.1	2.7	**< 0.001**
DIP (μM/L)	1.1	0.1	0.9	0.1	**< 0.001**
DIN (μM/L)	14.9	1.8	11.1	1.6	**< 0.001**
${\mathrm{NO}}_{2}^{-}$ (μM/L)	0.4	0	0.4	0	0.777
${\mathrm{NO}}_{3}^{-}$ (μM/L)	14.5	1.8	10.7	1.6	**< 0.001**

*Note:* Table shows mean and ± standard deviation (SD) of temperature (Celsius degrees, °C) and salinity (practical salinity unit, psu) measured using CTD, and nutrient concentrations (silicate, Si; dissolved inorganic phosphate, DIP; dissolved inorganic nitrogen, DIN; nitrite, ${\mathrm{NO}}_{2}^{-}$; and nitrate, ${\mathrm{NO}}_{3}^{-}$, μM/L) in the Northwest (NW‐GoA) and Southeast (SE‐GoA) regions. Significant *p*‐values for Welch two sample *t*‐test comparing the means between NW‐GoA and SE‐GoA are highlighted in bold.

### 3.2 Salmon Diets

**FIGURE 2:**
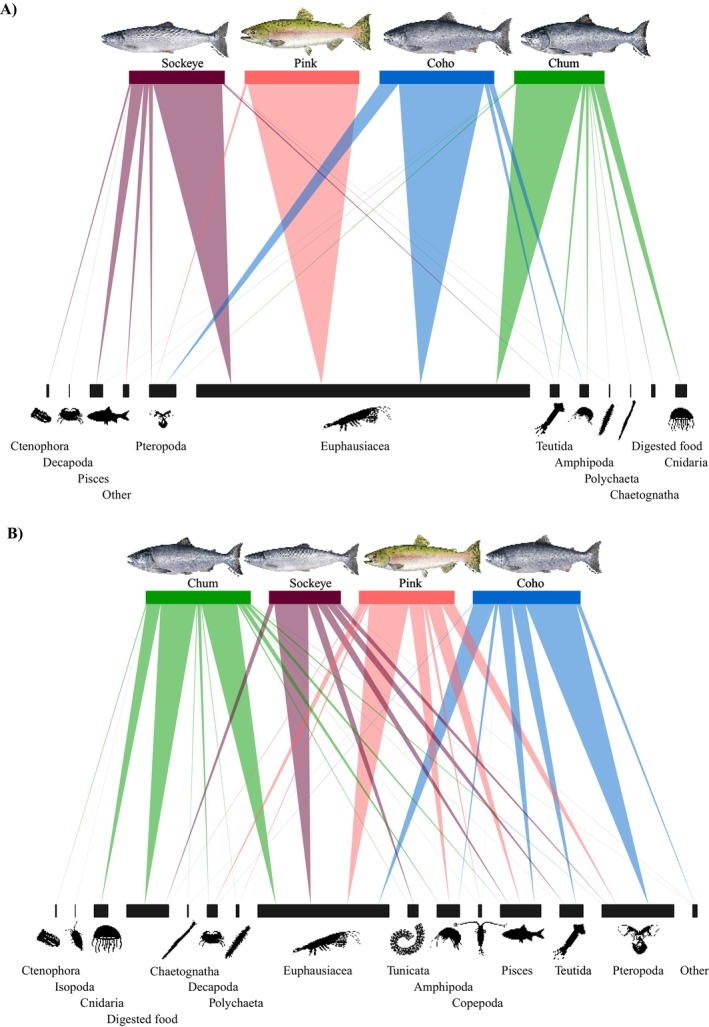
Bipartite network representing the predator–prey interactions among Pacific salmon in (A) the Northwest Gulf of Alaska and (B) the Southeast Gulf of Alaska. The bar width of salmon (predators, upper bars) represents the niche breadth of each salmon species. The bar width of prey (below) is proportional to the level of importance (by weight) to the diet of Pacific salmon, while the linkages' thickness is proportional to the importance (%) of each prey to each salmon species.

Consistent with environmental differences between the NW‐GoA and SE‐GoA, our data also revealed large spatial differences in Pacific salmon diets. The most important prey item (by weight) found in the stomachs of salmon sampled in the NW‐GoA was euphausiids, which comprised 98% of the prey remains in pink salmon, 75% in coho salmon, 61% in sockeye salmon, and 58% in chum salmon (Figure 2). The two Chinook salmon sampled in this region had consumed only fish. In the NW‐GoA, the next most important dietary items were pteropods (16.7%) in coho diets, cnidaria (9.6%) in chum diets, and fish (10.8%) in sockeye diets (Figure 2). In the SE‐GoA region, euphausiids' consumption decreased to 18%–34%, depending on the salmon species (Figure 2). Coho diets were dominated by pteropods (43%), followed by euphausiids (18%), squids (12%), and fish (11%). Sockeye diets were dominated by euphausiids (29%), followed by squid, fish, and salps (~7.6% each). Chum stomachs were dominated by digested food (31%), which we assumed included mostly gelatinous prey, with euphausiids making up 31% of the diet, followed by cnidaria (11.8%) and amphipods (4.5%). Finally, pink salmon diets included a high proportion of euphausiids (34%), fish (12%), amphipods (12%), and pteropods (11.6%, Figure 2).

### 3.3 Isotope Data

A total of 983 samples were collected and analyzed for carbon and nitrogen‐stable isotopes. Details on the number of samples collected in each subarea (NW‐GoA and SE‐GoA), their ẟ^13^C and ẟ^15^N values, and CN ratios are available at https://doi.org/10.21966/jxrd‐8g95 (Troina et al. 2024). In this study, we present the data for the identified meso‐predators, including species' sample size, stable isotope values (mean ± SD), estimated trophic position (mean ± SD) from the Post (2002) equation, and the mode and 95% credibility intervals from the Bayesian model (Table 2).

**TABLE 2:** Meso‐predator species collected in the northwest (NW‐GoA) and southeast (SE‐GoA) Gulf of Alaska during the International Year of the Salmon expedition in 2019.

Species	*N*	Min–Max length (mm)	ẟ^13^C′ (‰)		ẟ^15^N (‰)		CN		TrP (Post 2002)		TrP (Bayesian model)		
			Mean	SD	Mean	SD	Mean	SD	Mean	SD	Posterior mode	95% CI	
	**NW‐GoA**												
Salmon													
*Oncorhynchus gorbuscha* (pink)	1	372	−19.92	ND	11.56	ND	3.27	ND	3.58	ND	ND	ND	ND
*Oncorhynchus keta* (chum)	55	369–601	−20.88	0.51	10.42	0.87	3.28	0.18	3.24	0. 25	3.31	3.15	3.50
*Oncorhynchus kisutch* (coho)	11	317–415	−19.04	0.91	13.39	1.29	3.22	0.05	4.09	0.36	4.18	3.85	4.54
*Oncorhynchus nerka* (sockeye)^a^	8	332–397	−19.63	0.44	12.27	0.85	3.37	0.23	3.79	0.25	3.86	3.57	4.17
*Oncorhynchus nerka* (sockeye) (L)^b^	43	404–580	−20.25	0.47	11.37	0.76	3.45	0.24	3.52	0.23	3.6	3.41	3.79
*Oncorhynchus tshawytscha* (Chinook)	2	711–745	−19.58	0.32	12.42	0.47	4.61	0.45	3.83	0.14	ND	ND	ND
Non‐salmonid fish													
*Anotopterus nikparini*	1	360	−20.24	ND	10.42	ND	3.25	ND	3.24	ND	ND	ND	ND
*Diaphus theta*	20	50–70	−21.72	0.43	10.61	1.03	5.81	1.4	3.30	0.30	3.37	3.16	3.59
*Icichthys lockingtoni*	1	108	−20.43	ND	13.22	ND	3.57	ND	4.07	ND	ND	ND	ND
*Microstomus pacificus*	1	22	−20.51	ND	12.95	ND	5.75	ND	3.99	ND	ND	ND	ND
*Stenobrachius leucosparus*	22	27–86	−21.37	0.55	9.73	1.28	7.29	0.95	3.04	0.38	3.11	2.89	3.34
*Symbolophorous californensens*	1	98	−21.28	ND	11.67	ND	9.93	ND	3.61	ND	3.69	2	9.35
*Tarletonbeania crenularis*	74	42–78	−21.22	0.31	10.84	1.27	3.78	0.38	3.35	0.36	3.43	3.25	3.62
Squid													
*Abraliopsis felis*	5	34–42	−21.03	0.32	12.09	1.05	3.69	0.18	3.73	0.31	3.81	3.27	4.33
*Chiroteuthis calyx*	3	52–60	−21.75	0.33	14.45	0.78	2.62	0.43	4.43	0.23	4.49	3.21	5.84
*Gonatopsis borealis*	45	26–122	−21.22	0.34	8.77	1.7	3.63	0.2	2.75	0.51	2.82	2.63	3.04
*Gonatus madokai*	1	ND	−23.14	ND	6.82	ND	4.13	ND	2.18	ND	ND	ND	ND
*Gonatus onyx*	8	33–62	−21.28	0.55	11.46	1.89	3.66	0.4	3.37	0.58	3.61	3.22	4.02
*Onychyoteuthis borealjaponica*	4	75–201	−20.69	0.4	9.93	1.67	3.41	0.11	3.10	0.49	3.16	2.2	4.15
Jellyfish													
*Aequorea* sp.	5	21–105	−19.69	2.09	8.39	1.55	3.13	0.34	3.04	0.74	2.72	2.09	3.35
*Calycopsis simulans*	2	25–28	−20.81	0.21	11.53	0.57	4.23	0.06	4.46	0.34	3.65	2.61	4.72
*Phacellophoroa camtschatica*	7	80–400	−19.62	0.84	7.05	1.94	3.29	0.13	2.41	0.92	2.32	2	2.83
	**SE‐GoA**												
Salmon													
*Oncorhynchus gorbuscha* (pink)	15	283–397	−19.45	0.47	12.12	0.76	3.34	0.09	3.74	0.22	3.61	3.28	3.96
*Oncorhynchus keta* (chum)	26	380–582	−20.73	0.6	10.49	0.84	3.47	0.32	3.26	0.25	3.13	2.82	3.45
*Oncorhynchus kisutch* (coho)	45	337–490	−18.9	0.42	13.31	0.48	3.26	0.04	4.09	0.14	3.95	3.66	4.29
*Oncorhynchus nerka* (sockeye)	5	289–516	−20.37	0.31	11.5	0.76	3.5	0.4	3.56	0.23	3.42	2.94	3.89
*Oncorhynchus tshawytscha* (Chinook)	1	362	−18.88	ND	14.94	ND	3.29	ND	4.57	ND	ND	ND	ND
Non‐salmonid fish													
*Lestidiops ringens*	1	183	−20.13	ND	14.77	ND	3.4	ND	4.52	ND	ND	ND	ND
*Microstomus pacificus*	3	35–50	−20.93	1.12	12.26	1.58	6.04	1.62	3.62	0.41	3.65	2.57	4.7
*Squalus acanthias*	2	140–918	−19.17	1.21	14.43	1.24	6.62	5.03	4.42	0.37	4.24	2.02	8.07
*Symbolophorous californensens*	1	106	−20.38	ND	13.95	ND	6.27	ND	4.28	ND	ND	ND	ND
*Tarletonbeania crenularis*	29	27–84	−21.29	0.27	11.66	1.22	3.78	0.22	3.60	0.36	3.48	3.15	3.82
Squid													
*Abraliopsis felis*	2	37–43	−21.31	0.14	12.57	0.13	3.69	0.16	3.88	0.04	ND	ND	ND
*Gonatopsis borealis*	35	39–123	−21.14	0.44	8.7	1.83	3.56	0.12	2.73	0.54	2.6	2.24	2.93
*Gonatus onyx*	6	43–52	−21.42	0.26	9.57	1.18	3.61	0.11	2.99	0.35	2.86	2.33	3.35
*Onychyoteuthis borealjaponica*	30	7–183	−20.47	0.35	12.34	1.27	3.48	0.16	3.80	0.37	3.65	3.34	4.02
*Onykia robusta*	1	346	−22.71	ND	13.05	ND	7.4	ND	4.01	ND	ND	ND	ND
*Watasenia scintillans*	2	35–37	−21.07	0.66	14.28	1.31	3.61	0.08	4.38	0.39	ND	ND	ND
*Taonius borealis*	3	ND‐600	−20.45	0.77	13.66	0.84	3.42	0.14	4.08	0.12	4.05	3.37	4.79
Jellyfish													
*Aequorea* sp.	2	35–150	−17.25	0.07	11.01	0.83	2.89	0.05	4.29	0.40	ND	ND	ND
*Aurelia labiata*	3	80–230	−19.79	0.7	8.14	1.08	3.32	0.03	3.01	0.52	2.44	2	4.11
*Calycopsis simulans*	1	25	−20.73	ND	10.67	ND	3.93	ND	4.13	ND	ND	ND	ND
*Phacellophoroa camtschatica*	4	180–420	−18.99	0.35	10.8	1.34	3.28	0.12	4.19	0.64	3.22	2.37	4.11

*Note:* Data include the number of samples (*N*), min and max length (mm), ẟ^13^C′ (lipid corrected), ẟ^15^N, carbon:nitrogen (CN) ratios, and estimated trophic position (TrP). TrP is presented as mean ± standard deviation (SD) values estimated per species using the equation presented in Post (2002), and the posterior mode with the 95% credibility intervals (CI) using the Bayesian framework. ND (no data) is used to indicate that the value could not be estimated due to the small sample size. For details on the complete dataset, we refer to Troina et al. (2024).

^a^ *Oncorhynchus nerka* (sockeye salmon) 300–400 mm size class.

^b^ *Oncorhynchus nerka* (sockeye salmon) (L) size class with specimens larger than 400 mm.

### 3.4 Trophic Position

The trophic position of Pacific salmon was consistent in both areas, ranging between 3.2 in chum salmon and 4.2 in coho salmon. Coho and Chinook salmon had the highest TrP among the salmonid species, followed by pink and sockeye salmon, with chum having the lowest TrP (Table 2). The TrP of all salmon species, except for Chinook salmon, were slightly higher in the NW‐GoA, although the probability of overlap between the posterior distributions was relatively high (> 0.69, Appendix S2), indicating that the posterior distributions of estimated TrP were not different. Coho salmon showed a high probability of occupying the highest TrP among the salmon species, in both areas. Conversely, the probability of the posterior TrP of chum salmon being higher than the other salmon species was low (between 0 and 0.24, Table 3) in both regions, indicating the species' lowest TrP among the salmon species. In the NW‐GoA, small sockeye salmon had slightly higher trophic positions (TrP = 3.8 ± 0.25) than the larger sockeye (TrP = 3.5 ± 0.23, Table 2).

**TABLE 3:** Comparison of the posterior distributions of trophic position (TrP) between salmon and non‐salmon meso‐predators within each region: Northwest (NW‐GoA) and southeast (SE‐GoA) Gulf of Alaska.

	*O. gorbuscha*		*O. keta*		*O. kisutch*		*O. nerka*		*O. nerka* (L)	
NW‐GoA	Overlap	Prob > TrP	Overlap	Prob > TrP	Overlap	Prob > TrP	Overlap	Prob > TrP	Overlap	Prob > TrP
*Oncorhynchus keta* (chum)	ND	ND	—	—	0.02	1.00	0.15	0.99	0.35	0.98
*Oncorhynchus kisutch* (coho)	ND	ND	0.02	0.00	—	—	0.68	0.00	0.16	0.00
*Oncorhynchus nerka* (sockeye)^1^	ND	ND	0.15	0.00	0.68	0.91	—	—	0.62	0.07
*Oncorhynchus nerka* (sockeye) (L)^2^	ND	ND	0.35	0.02	0.16	1.00	0.62	0.93	—	—
*Diaphus theta*	ND	ND	0.96	0.35	0.05	1.00	0.25	0.97	0.57	0.94
*Stenobrachius leucosparus*	ND	ND	0.63	0.92	0.01	1.00	0.06	1.00	0.10	1.00
*Tarletonbeania crenularis*	ND	ND	0.83	0.19	0.06	1.00	0. 29	0.93	0.70	0.89
*Abraliopsis felis*	ND	ND	0.41	0.04	0.71	0.91	0.97	0.24	0.90	0.19
*Chiroteuthis calyx*	ND	ND	0.18	0.03	0.79	0.21	0.51	0.05	0.29	0.04
*Gonatus onyx*	ND	ND	0.82	0.31	0.38	0.98	0.71	0.53	0.86	0.71
*Onychyoteuthis borealijaponica*	ND	ND	0.76	0.64	0.34	0.96	0.50	0.85	0.63	0.84
*Aequorea* sp.	ND	ND	0.41	0.95	0.12	1.00	0.19	0.98	0.27	0.98
*Calycopsis simulans*	ND	ND	0.54	0.24	0.57	0.91	0.69	0.48	0.69	0.42

SE‐GoA	Overlap	Prob > TrP	Overlap	Prob > TrP	Overlap	Prob > TrP	Overlap	Prob > TrP		
*Oncorhynchus gorbuscha* (pink)	—	—	0.40	0.02	0.61	0.93	0.91	0.25	—	—
*Oncorhynchus keta* (chum)	0.40	0.98	—	—	0.08	1.00	0.84	0.86	—	—
*Oncorhynchus kisutch* (coho)	0.61	0.07	0.08	0.00	—	—	0.57	0.03	—	—
*Oncorhynchus nerka* (sockeye)	0.91	0.75	0.84	0.14	0.57	0.97	—	—	—	—
*Microstomus pacificus*	0.90	0.46	0.73	0.22	0.80	0.76	0.90	0.31	—	—
*Tarletonbeania crenularis*	0.93	0.72	0.62	0.07	0.40	0.98	0.99	0.43	—	—
*Taonius borealis*	0.70	0.09	0.33	0.03	0.95	0.38	0.60	0.05	—	—
*Gonatus onyx*	0.37	0.99	0.83	0.83	0.14	1.00	0.62	0.95	—	—
*Onychyoteuthis borealijaponica*	0.99	0.40	0.32	0.01	0.72	0.89	0.86	0.19	—	—
*Phacellophoroa camtschatica*	0.75	0.83	0.91	0.40	0.47	0.94	0.93	0.68	—	—
*Hormiphora cucumis*	0.56	0.94	0.90	0.65	0.29	0.99	0.79	0.86	—	—

*Note:* Overlap was calculated using the Bhattacharyya coefficient and indicates the probability of overlap between two posterior distributions. “Prob” indicates the probability of a salmon species (column) having a posterior trophic position higher than or equal to the species in each row. ND (no data) is used to indicate that the value could not be estimated due to the small (*N* < 3) sample size.

**FIGURE 3:**
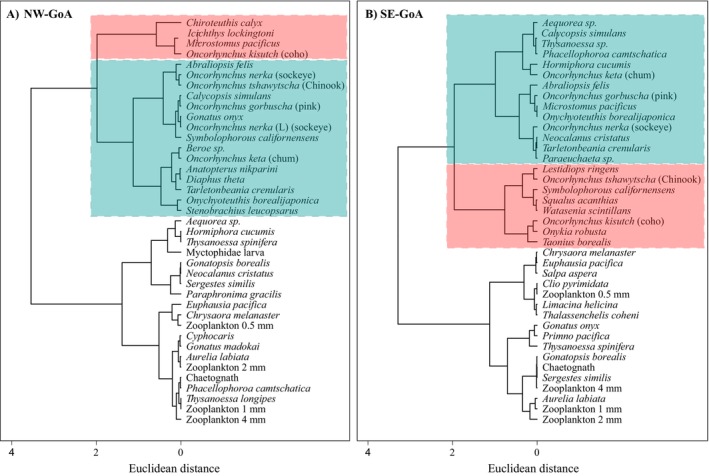
Hierarchical cluster analysis for the (A) Northwest (NW‐GoA) and (B) Southeast (SE‐GoA) Gulf of Alaska food webs, based on species estimated trophic positions. The red box delineates the higher trophic level and the blue box delineates lower trophic level meso‐predators. Lower trophic level groups are not highlighted.

Furthermore, our analysis identified several species of squid, myctophids, and jellyfish that had similar trophic positions to those of salmon (Figure 3). The Bhattacharyya coefficient, applied to calculate the probability of overlap between the posterior TrP estimates of every pair of species showed a high degree of overlap in the trophic position of some non‐salmonid species and Pacific salmon. Specifically, the myctophids *Diaphus theta*, *Tarletonbeania crenularis*, the flatfish *Microstomus pacificus*, and the cephalopods *A. felis*, *T. borealis*, *C. calyx*, *Gonatus onyx* and *Onychyoteuthis borealijaponica* had high probabilities of overlap in the posterior TrP estimates with chum, sockeye, and pink salmon (Table 3).

### 3.5 Isotopic Niche Patterns

**FIGURE 4:**
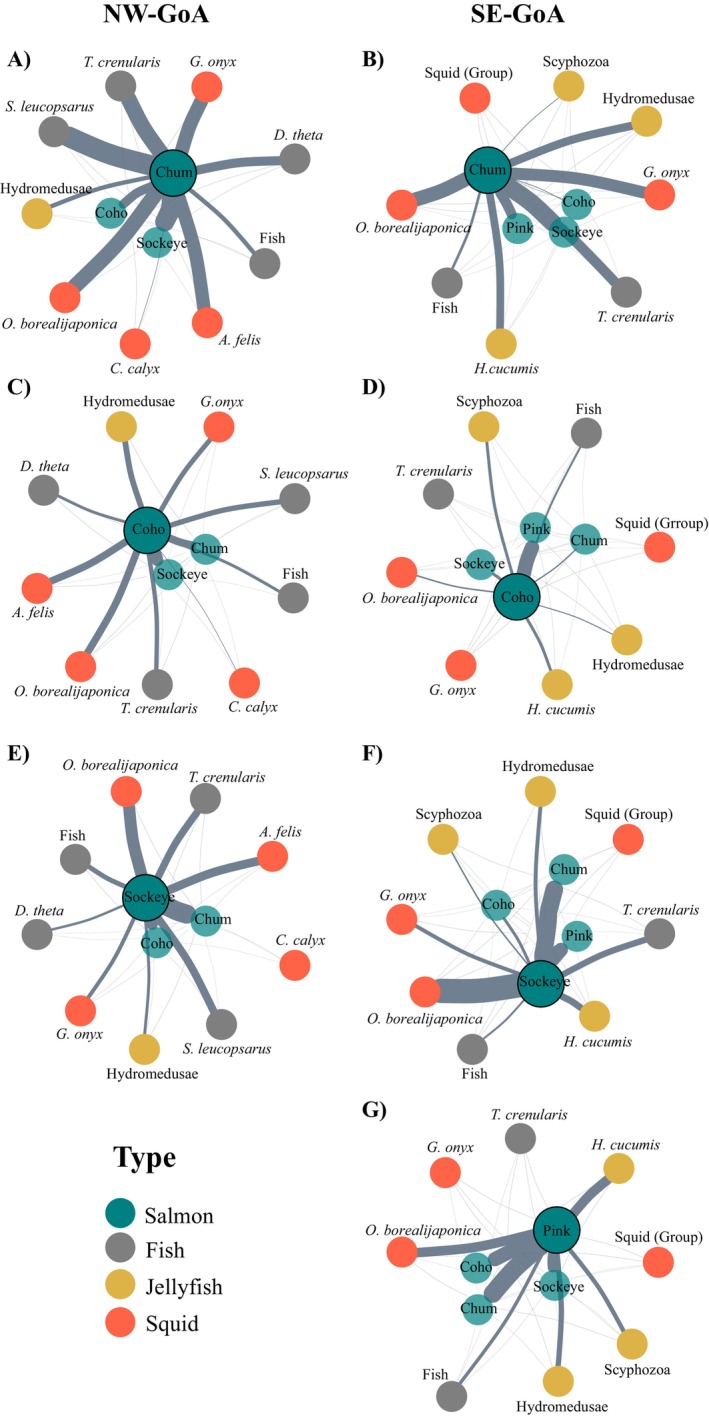
Network plots representing the isotopic niche overlap between (A and B) Chum, (C and D) Coho, (E and F) Sockeye, and (G) Pink salmon and the other meso‐predators in the Northwest (NW‐GoA, left side) and Southeast (SE‐GoA, right side) Gulf of Alaska. The thickness of the edges is proportional to the percentage of the niche area overlap. Absolute values are shown in Appendix S3.

The level of isotopic niche overlap among Pacific salmon species differed between NW‐GoA and SE‐GoA. In the NW‐GoA, the chum salmon isotopic niche overlapped by 46% with the large sockeye, 21% with small sockeye, and 18% with coho salmon. The isotopic niche overlap between coho salmon and the large sockeye salmon was 23%, and 45% with small sockeye (Figure 4, Appendix S3). In the SE‐GoA, pink salmon isotopic niche overlapped by ~30% with coho and sockeye salmon, and 21% with chum. Chum salmon isotopic niche overlapped by 42% with sockeye salmon, and only 2% with coho salmon (Figure 4, Appendix S3). The isotopic niche area of coho salmon overlapped by 30% with that of pink salmon and 6% with sockeye (Figure 4).

Considering the interaction between salmon and non‐salmon species, in both areas, chum salmon showed considerable isotopic niche overlap with most species, including the myctophids *Stenobrachius leucosparus* (34%), *T. crenularis* (24%–35%), and the squids *O. borealijaponica* (28%–32%), *G. onyx* (21%–28%), and *A. felis* (27%) (Figure 4A,B, Appendix S3). Sockeye salmon isotopic niche overlapped only with the squid *O. borealijaponica* and had an approximately twofold increase from the NW‐GoA (25%) to the SE‐GoA (51%, Figure 4E,F). In the SE‐GoA, pink salmon had low isotopic niche overlap with the non‐salmonid species, except for the squid *O. borealijaponica* and the ctenophore *Hormiphora cucumis* (20%, Figure 4G). Lastly, our data indicated low isotopic niche overlap between coho salmon and the other non‐salmon species in both NW‐GoA (< 15%) and SE‐GoA (< 6%), (Figure 4C,D; Appendix S3).

### 3.6 Biomass Spatial Distribution

**FIGURE 5:**
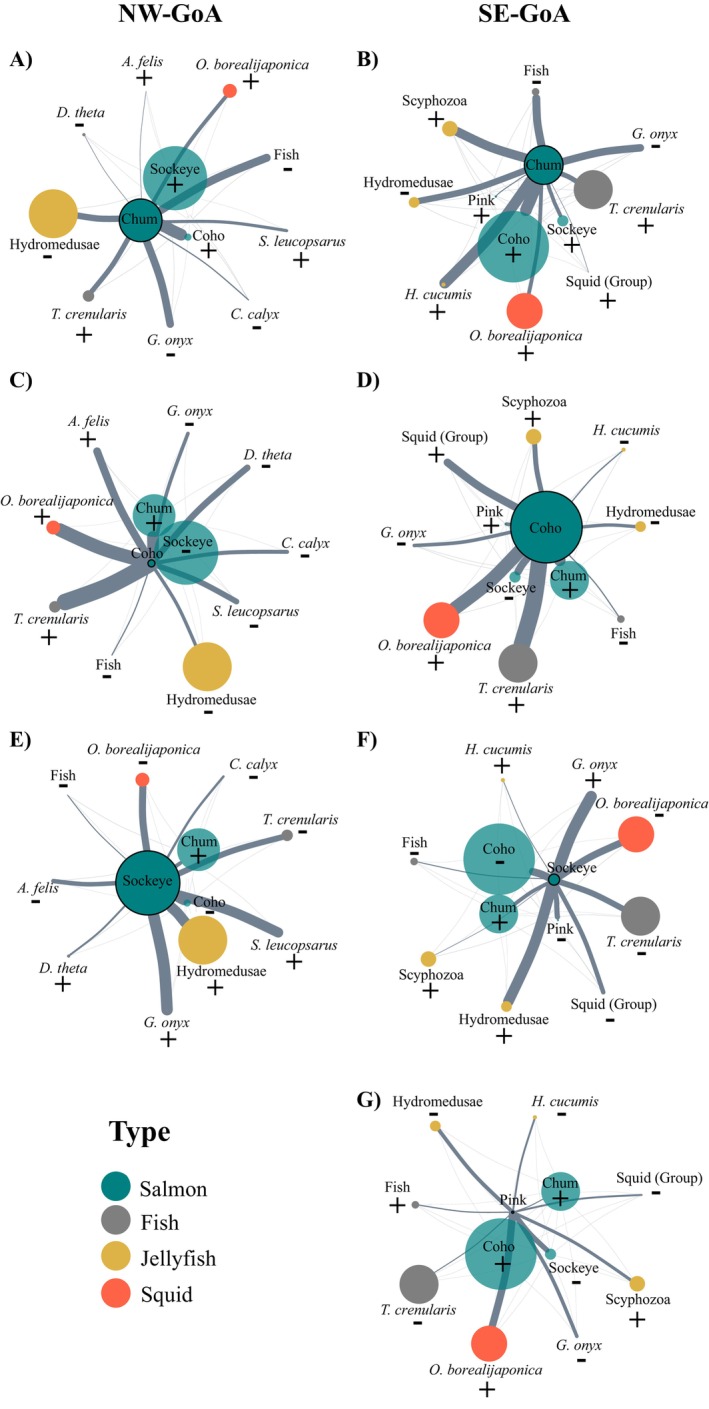
Biomass correlation between (A and B) Chum, (C and D) Coho, (E and F) Sockeye, and (G) Pink salmon and the other meso‐predators in the Northwest (NW‐GoA, left side) and Southeast (SE‐GoA, right side) Gulf of Alaska. Nodes are colored according to the group, and node size is proportional to species biomass, + and − signs indicate positive and negative correlation, respectively, and the thickness of the edges indicates the strength of the correlation (correlation coefficient *r*): The thicker the edge, the stronger is the correlation between paired species. Absolute values for the biomass correlation coefficient and estimated biomass for each meso‐predator species are shown in Appendices S3 and S4, respectively. The correlation was calculated based on the entire dataset.

Using estimates of species‐ and group‐specific biomass, we observed positive and negative spatial correlations among both salmonid and non‐salmonid species (Appendix S1). The Pearson correlation coefficient indicated a positive correlation between chum salmon and coho salmon (0.50), *H. cucumis* (0.46), and squid *O. robusta* (0.44) and *Gonatus* sp. (0.43), and a negative correlation with *Microstomus pacificus* (−0.34), *G. onyx* (−0.32), and *C. simulans* (−0.33, Figure 5A,B). Coho salmon had a high positive correlation with the myctophid *T. crenularis* (0.71), the squids *Gonatopsis borealis* (0.58), *O. borealijaponica* (0.57), and *O. robusta* (0.53, Figure 5C,D). Sockeye salmon biomass was positively correlated with those of the squids *G. madokai* (0.55) and *G. onyx* (0.49), the myctophid *S. leucosparus* (0.43), and jellyfish (Figure 5E,F). On the other hand, sockeye and *O. borealijaponica* spatial distributions were negatively correlated (−0.30). Pink salmon biomass distribution correlated positively with those of *Symbolophorus californiensis* (0.70) and *O. borealijaponica* (0.31, Figure 5G). In addition, squids, myctophids, and sockeye salmon were primarily caught during the night trawls in both regions, while pink and chum salmon were mostly caught at day trawls (Appendix S4).

**FIGURE 6:**
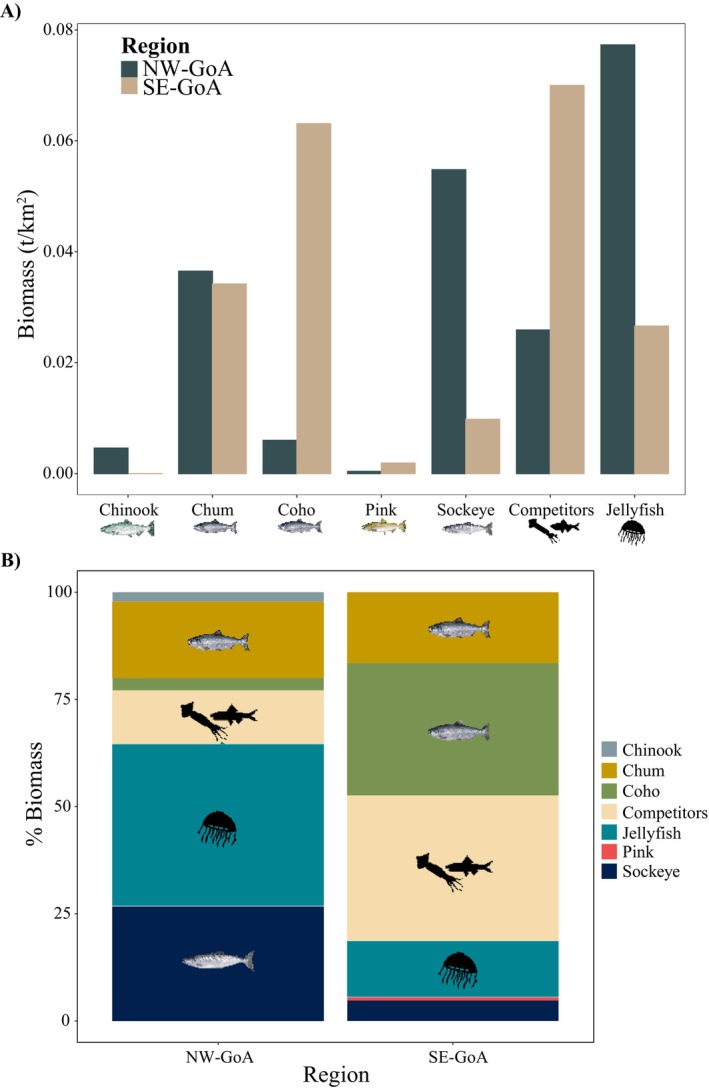
(A) Average biomass in tons (t) per km^2^ of the five Pacific salmon species, jellyfish, and potential competitors collected in the Northwest (NW‐GoA) and the Southeast (SE‐GoA) Gulf of Alaska in the winter of 2019 and (B) proportional contribution (%) of the different salmon species, competitors and jellyfish to the total biomass of organisms sampled in each area. Jellyfish biomass shown here only accounts for the jellyfish species that had similar trophic positions to Pacific salmon, as identified by our cluster analysis.

Overall, biomasses were high for jellyfish (hydromedusae), sockeye and chum salmon in the NW‐GoA (Figures 5 and 6), while in the SE‐GoA biomasses were high for the squid *O. borealijaponica*, the myctophid *T. crenularis* , coho and chum salmon (Figures 5 and 6). The summed biomasses of all the potential competitor species (Figure 6), as defined by the high isotope niche overlap with one or more salmon species (NW‐GoA: *A. felis* , *D. theta* , *G. onyx* , *O. borealijaponica*, *S. leucosparus*, and *T. crenularis* ; SE‐GoA: *G. onyx* , *H. cucumis* , *O. borealijaponica*, *and T. crenularis*) was 0.03 t/km^2^ in the NW‐GoA (10,577 tons for the entire surveyed area of 408,445 km^2^), while in the SE‐GoA competitor biomass was 0.07 t/km^2^ (20,459 tons for the entire surveyed area of 292,166 km^2^, Appendix S5).

## 4 Discussion

Our isotopic data revealed that Pacific salmon and non‐salmonid meso‐predators in the Gulf of Alaska used isotopically similar resources during winter. While competition among salmonid species has been recognized in the Eastern North Pacific, especially between pink salmon and the other salmon species (Batten, Ruggerone, and Ortiz 2018; Northern Hemisphere Pink Salmon Expert Group 2023; Ruggerone et al. 2023), our findings highlight that the high degree of isotopic niche overlap observed creates a potential for competition that may take place between Pacific salmon and non‐salmonid meso‐predators during winter. In addition to identifying potential trophic interactions among North Pacific meso‐predator species, we provide valuable insights into the trophic ecology of lesser‐known pelagic fish and cephalopod species in this region.

### 4.1 Trophic Ecology of North Pacific Meso‐Predators

Information on the life history and feeding behavior of open ocean fish and cephalopods is limited in the Northeast Pacific Ocean, mostly due to the challenges involved in collecting wild specimens in the high seas. Here, we discuss the patterns observed in the stable isotope values of the different species to improve our understanding of their feeding ecology and trophic interactions. Our data indicated no significant spatial differences in the trophic positions of the meso‐predator species sampled across both areas, contrary to our first hypothesis that they would. In both areas, our hierarchical cluster analysis identified two main trophic groups comprised of meso‐predator species in the Northeast Pacific Ocean, based on these species' trophic positions. The higher trophic level meso‐predators identified were the cartilaginous fish *S. acanthias* , the fishes *Lestidiops ringens* , *Icichthys lockingtoni* , *S. californiensis* , Chinook and coho salmon, and the squids *C. calyx* , *T. borealis* , *O. robusta* , and *W. scintillans* (Figure 3). Our isotopic data suggested that these species are tertiary consumers with estimated trophic positions around or greater than 4, agreeing with estimates shown on fishbase.org (Froese and Pauly 2023). The only exception was the lanternfish *S. californiensis* , for which food items (Froese and Pauly 2023) suggest a relatively lower TrP (~3.1 ± 0.3) than our estimates here. In the case of Chinook and coho salmon, previous works based on stable isotopes and stomach content analysis have consistently reported a diet primarily composed of large micronekton such as fish and squids (Kaeriyama et al. 2004; Qin and Kaeriyama 2016). The analysis of the stomachs of the three Chinook collected in 2019 revealed the presence of squids (*Okutania anonycha*) and fish (data not shown). Coho salmon consumed mainly euphausiids in the NW‐GoA. However, in the SE‐GoA their diet consisted of euphausiids, pteropods, cephalopods, and fish. Generally, juvenile squids feed on small planktonic crustaceans while shifting to a diet based on higher trophic level prey, such as myctophids and other squids in adulthood (Boyle and Rodhouse 2005). Thus, considering a diet based on macrozooplankton and nektonic organisms, TrP greater than 4.0 would be expected and is supported by the stable isotope data analyzed here.

The second group identified in the cluster analysis included the relatively lower trophic level meso‐predator species. Among the Pacific salmon were chum (lowest trophic position among salmon), sockeye, and pink salmon. Their estimated trophic position around 3–3.5 indicates that these are omnivorous secondary consumers. Indeed, sockeye and pink salmon may switch between zooplankton and micronekton prey (Graham, Pakhomov, and Hunt 2021; Kaeriyama et al. 2004, 2000; Qin and Kaeriyama 2016; Volkov 2014, 2016; Chuchukalo 2006), whereas chum salmon diet includes crustacean zooplankton but is often dominated by a large proportion of gelatinous prey (Graham, Pakhomov, and Hunt 2021; Kaeriyama et al. 2004; Qin and Kaeriyama 2016; Volkov 2014, 2016; Chuchukalo 2006). Large‐scale dietary studies within the North Pacific Ocean have demonstrated that chum, pink, and sockeye salmon feeding habits vary according to spatial gradients in prey availability (Graham, Pakhomov, and Hunt 2021; Kaeriyama et al. 2004, 2000; Qin and Kaeriyama 2016; Zavolokin 2011). While chum salmon usually occupy the lowest trophic position among Pacific salmon species, regardless of whether they are feeding on crustacean or gelatinous zooplankton (Qin and Kaeriyama 2016), sockeye and pink salmon may occupy different trophic positions within food webs when switching between planktonic and nektonic prey (Graham, Pakhomov, and Hunt 2021; Qin and Kaeriyama 2016; Zavolokin 2011). In addition, pink salmon tend to consume a greater diversity of prey when the abundance of intra‐ and interspecific counterparts is high (Kaeriyama et al. 2000; Qin and Kaeriyama 2016). Although our isotope data did not show evidence for gradients in sockeye and pink salmon trophic positions between the NW‐GoA and SE‐GoA, all Pacific salmon species consumed a greater variety of prey in the latter region. Such gradients in salmon feeding behavior in the North Pacific highlight how food web dynamics may be affected by changes in ocean conditions, prey availability, and interspecific trophic interactions. In turn, it is important to assess how these dietary shifts may affect these species' growth rates and nutritional health. Lastly, the slightly higher trophic position estimated for the small sockeye salmon in the NW‐GoA, in comparison to the larger size‐class, likely represents a fraction of the ẟ^15^N incorporated during the neritic phase, as nitrogen isotopic baselines tend to be higher in coastal waters (Espinasse et al. 2020; Troina et al. 2020).

Regarding the non‐salmonid fish species analyzed here, our isotope data estimated trophic positions between 3.1 and 3.6 for the myctophids *S. leucopsarus* , *T. crenularis* , and *D. theta* (Table 2), suggesting they consume a mixture of herbivorous and carnivorous plankton. Dietary studies in the North Pacific Ocean have reported euphausiids ( *E. pacifica* ), a variety of copepods, and some amphipods in the stomachs of *S. leucopsarus* (Cailliet and Ebeling 1990; Moku et al. 2000; Pearcy, Lorz, and Peterson 1979; Tyler and Pearcy 1975; Balanov 1994; Balanov and Gorbatenko 1995; Balanov, Gorbatenko, and Efimkin 1991, Balanov, Gorbatenko, and Gorelova 1994). Overall, euphausiids are more frequent in the diet of large specimens caught in surface waters, while copepods dominate the diet of specimens caught at greater depths (Pearcy, Lorz, and Peterson 1979). However, *S. leucopsarus* seems to target euphausiids when this prey type is abundant (Cailliet and Ebeling 1990). Similarly, the diet of *T. crenularis* and *D. theta* includes euphausiids ( *E. pacifica* ), copepods, and amphipods (Moku et al. 2000; Tyler and Pearcy 1975). The most common copepod species reported in their stomachs were *Neocalanus* spp. and *Metridia pacifica* (Moku et al. 2000; Tyler and Pearcy 1975). These large calanoid copepods are not exclusively herbivores, feeding on both phytoplankton and microzooplankton (Dagg 1993; Gifford 1993; Kobari, Shinada, and Tsuda 2003). Considering this, a trophic position above 2 would be expected, and this was confirmed by our isotope data (Troina et al. 2024). Accordingly, planktivorous meso‐predators that feed on these copepod species would have a trophic position around 3.5. The diet of the myctophid *S. californiensis* is dominated by calanoid copepods, but adults also take small or juvenile cephalopods and euphausiids (Sassa and Takasuka 2019; Takagi et al. 2009). Thus, the estimated trophic position for the two specimens collected in this study (TrP 3.7–4.0) suggests that they may opportunistically consume higher trophic level prey (such as cephalopods). Another fish species with a small sample size (*N* = 1) was the daggertooth *Anotopterus nikparini* , with estimated trophic position of 3.3. This species is known to be able to target larger, thus higher trophic level prey (Welch et al. 1991; Welch and Pankhurst 2001) and potentially impact Pacific salmon, at least in the northwest Pacific (Melnikov 1997). However, the specimen sampled here was small (36 cm), and thus likely consumed smaller, lower trophic level prey. Continued research and increased sample size are required for more robust inference of these species' feeding patterns during winter in the Gulf of Alaska.

As was the case for most of the meso‐predator species sampled in the North Pacific open ocean during the IYS 2019 winter expedition, there is limited specific information about the feeding ecology and behavior of the squids *A. felis* and *G. onyx* . Our isotope data pointed to a trophic level ranging between 3.6 and 3.8 for *A. felis* , and 2.8 (in the SE‐GoA) and 3.6 (NW‐GoA) for *G. onyx* (Table 2). In the case of *G. onyx* , specimens sampled in Monterey Bay have been found to consume mesopelagic fish, mainly *S. leucopsarus*, although cannibalism was particularly high (Hoving and Robison 2016). If *G. onyx* were feeding on *S. leucopsarus* in the open ocean during the period of our sampling, we would expect them to have had relatively higher TrP than these myctophids. Instead, our isotope data suggested that these squids were feeding on lower trophic level prey, at least during winter months in the high seas. Isotope data for the squid *O. borealijaponica* revealed a mean trophic position of 3.2 and 3.7 in the NW‐GoA and SE‐GoA, respectively, supporting an omnivorous diet based on primary and secondary consumers. Information on this species' feeding ecology is scarce. Earlier studies have reported mostly empty stomachs, and a few specimens with small fish and squid (Kubodera 1986; Okutani and Murata 1983). More recent surveys have detected a high proportion of fish, in addition to amphipods, euphausiids ( *E. pacifica* ), and decapods (Pakhomov et al. 2023).

The lack of dietary information for many of these open ocean meso‐predator species prevents any further comparisons with the trends observed here. The challenges involved with sampling open ocean predators lie beyond the difficult access to specimens due to distance from the coast. In the case of cephalopod species, for example, reliable dietary observations are rare as these are fast‐moving animals often capable of avoiding nets (Piatkowski, Pierce, and Morais Da Cunha 2001; Rodhouse 1990), and, when collected, stomachs are often empty due to regurgitation after the specimen is caught (Okutani and Murata 1983). Additionally, the identification of prey may be hindered due to selective feeding on soft parts of prey and active avoidance of prey's hard parts (Boyle and Rodhouse 2005). Given the challenges of obtaining dietary data for species that inhabit open ocean pelagic ecosystems, the analysis of stable isotopes in body tissue provides a valuable method for assessing these species' feeding ecology. Efforts should be made to continue monitoring the North Pacific high seas, particularly during winter months, to allow for a better understanding of these species' trophic ecology and food web dynamics.

### 4.2 Regional Differences in Meso‐Predator Trophic Ecology and Potential for Competition

The level of isotopic niche overlap between Pacific salmon species differed between those that were sampled in the NW‐GoA and those from the SE‐GoA. Our analysis revealed a larger overlap in the isotopic niche among the three Pacific salmon species (coho, sockeye, and chum) sampled in the NW‐GoA, while in the SE‐GoA isotopic overlap was significantly lower between coho salmon and both chum (2%) and sockeye (6%) salmon. These findings were consistent with the dietary data, which indicated that all three species consumed a substantial amount of euphausiids in the NW‐GoA (Figure 2). On the other hand, coho salmon shifted their diet from being predominantly composed of euphausiids in the NW‐GoA (75%) to being dominated by pteropods (43.8%) in the SE‐GoA. The importance of euphausiids decreased to 18.6% in the SE‐GoA, with fish and squids accounting for most of the remaining prey found in coho salmon' stomachs. Therefore, the observed high dietary and isotopic overlap in the NW‐GoA seemed to be primarily driven by the consumption of euphausiids, while a more variable diet in the SE‐GoA was associated with lower levels of isotope niche overlap between coho salmon and both chum and sockeye salmon (Figure 4). Thus, although these two methods provided dietary information representative of different timescales (i.e., weeks to months for stable isotopes vs. hours for diet data), there was overall agreement in both assessments.

The high isotopic niche overlap between coho and the small sockeye salmon in the NW‐GoA (45%) should be viewed with caution, due to the potential influence of neritic isotope values in smaller first winter sockeye salmon. Future research applying compound‐specific isotope analysis could help to disentangle the effects of trophic position and shifted ẟ^15^N baselines (Troina et al. 2021) and would aid the understanding of trends in the foraging ecology and trophic interactions among North Pacific meso‐predators and how the different size‐classes of Pacific salmon are affected.

Furthermore, the isotopic niche overlap between coho salmon and the non‐salmonid species was found to be extremely low in the NW‐GoA and virtually non‐existent in the SE‐GoA. This result was not surprising considering the relatively higher trophic position of coho salmon in the North Pacific food webs (see Section 4.1). In the SE‐GoA, coho salmon were found to predominantly consume pteropods, specifically *Clio pyramidata* , during the 2019 expedition. Isotopic data for the single sample of *C. pyramidata* obtained in the SE‐GoA yielded a TrP of 1.6 (Troina et al. 2024), supporting this species' known herbivorous feeding habit (Hunt et al. 2008; Phleger et al. 2001). The high TrP observed in coho salmon is likely a result of their consumption of fish and squid prey, which increases their overall trophic level. This unique feeding behavior of coho salmon in the SE‐GoA, involving both pteropods and fish/squid prey, may provide them with certain advantages, such as reduced interspecific competition for food. This, in addition to the species preference for higher temperature waters (Myers et al. 2016), might help to explain coho's relatively higher biomass in the SE‐GoA in comparison to the NW‐GoA.

The high overlap in isotopic niche between salmon and some of the non‐salmonid meso‐predator species suggests that these important components of the North Pacific food webs may compete for food during the winter months. In particular, both chum and sockeye salmon consistently showed substantial isotopic niche overlap with different squid and myctophid species in both the NW‐GoA and SE‐GoA. However, the level of isotopic niche overlap between the pairs of meso‐predator species varied between these two regions. For example, sockeye salmon were predominantly caught in the NW‐GoA, where this species exhibited intermediate levels of isotopic niche overlap with various non‐salmonid species (see Figure 4E). Although there were a substantial number of interactions (i.e., a number of species with some level of isotopic niche overlap), the biomasses of these non‐salmonid species were relatively low in the NW‐GoA.

By contrast, in the SE‐GoA, the number of non‐salmonid species with whom sockeye salmon's isotopic niche overlapped decreased significantly. However, the intensity of the isotopic niche overlap with the squid *O. borealijaponica* increased to 51% (Figure 4F). Squids and myctophids were primarily caught during the night trawls, which support diel vertical migration by these groups. The fact that sockeye salmon were also mostly caught at night demonstrated high similarity in habitat use both temporally and spatially (i.e., the use of surface waters at night). Thus, our data demonstrated that there is a high isotopic niche overlap between sockeye salmon and *O. borealijaponica* in the SE‐GoA, with high spatio‐temporal similarity in habitat preferences. However, the negative correlation (−0.3, Appendix S1) between these two species' biomass distribution may point to a context‐dependent interaction, in which the dominant competitor varies according to temperature gradients (Reese and Harvey 2002; Reeves, Everest, and Hall 1987). In this case, it seems that sockeye salmon was the dominant competitor in the colder waters of the NW‐GoA, while the squid *O. borealijaponica* was dominant in the warmer transitional waters of the SE‐GoA. Thus, these two species may be using similar resources and occupying equivalent ecological niches along gradients of temperature regimes. We highlight that our small sample size may have influenced the *p*‐values of the spatial correlation analyses, which were not significant (*p* > 0.05, Appendix S1). Therefore, we recommend further research to allow for stronger assessments of these relationships.

The isotopic niche overlap trends between chum salmon and the other meso‐predators contrasted with those observed in sockeye salmon. In the NW‐GoA, chum salmon exhibited high isotopic niche overlap with a greater number of species. In contrast, in the SE‐GoA, there were fewer species with intermediate (24%–28%) to high (32%–42%) levels of isotopic niche overlap with chum salmon. Notably, chum salmon showed significant isotopic niche overlap not only with other salmonid species but also with several squid and myctophid species in both NW‐GoA and SE‐GoA. This suggests that across the study area and its varying ocean conditions, chum salmon may play similar ecological roles and potentially compete for resources with different meso‐predator species during the winter. This contrasts with a previous isotope study that suggested that chum salmon use different trophic pathways from other salmonids, attributed to this species consumption of gelatinous prey (Welch and Parsons 1993). Accordingly, the authors found little potential for competition for food with the other Pacific salmon species.

Chum salmon were caught throughout the Gulf of Alaska with little variation in biomass between the two identified regions (NW‐GoA = 0.036 t/km^2^; SE‐GoA = 0.034 t/km^2^, Appendix S5), thus potential competition does not seem to be affecting this species longitudinal distribution. Most chum salmon were caught during the day (Appendix S4) while squids and myctophids were mainly caught at night. This could be linked to chum salmon's ability to consume gelatinous prey, which is also found in surface waters during the day and was the dominant prey found in chum salmon stomachs. The use of surface waters during the day could implicate small‐scale spatial niche segregation between chum and the other meso‐predator species. However, small‐scale spatial (water‐depth) or temporal (diel) habitat segregation might not eliminate exploitation competition if limited resources are shared between these species. The high isotopic niche overlap provides support for the use of isotopically similar prey which could lead to potential competition if food resources are limited. The high frequency of skinny chum salmon in the Gulf of Alaska during the 2019 winter (Troina et al. 2024; Urawa et al. 2022), as revealed by Fultons condition factor (Ricker 1975), could be indicative of competition affecting chum salmon nutritional health. Long‐term assessment (1925–2015) of weight trends in chum salmon returning from the North Pacific high seas have indicated a decreasing weight over time (Ruggerone and Irvine 2018). In addition, the authors found a negative correlation between chum salmon weight and the abundance of Pacific salmon, suggesting density‐dependent competition. Although relatively higher temperatures in the Gulf of Alaska have been suggested as an explanation for this species poor condition and low weight/lipid content in 2019 (Urawa et al. 2022), they could also be a result of competition between chum salmon and the other meso‐predator species during winter months. Future research should assess chum salmon's nutritional health and growth, for example, using fatty acids, in addition to applying food web models to evaluate the impact of competition for food on chum salmon condition and biomass trends.

Pink salmon exhibited a high isotopic niche overlap with all other salmon species sampled in the SE‐GoA. Although the stomach content analysis revealed a greater diversity of prey types consumed by Pacific salmon in the SE‐GoA, overall, chum, sockeye, and coho salmon consumed similar prey to pink salmon, albeit with variations in the relative importance of each prey type among these species. For instance, while euphausiids comprised approximately 30%–35% of the diet of all salmon species in the SE‐GoA, pteropods were important prey items for coho and pink salmon, and gelatinous prey was important in the diet of chum and pink salmon. Other prey items that were consumed in different proportions by these salmon species included small fish and amphipods. These dietary overlaps likely contributed to the significant isotopic niche overlap observed between pink salmon and the other Pacific salmon species. Thus, it appears that pink salmon display a generalist feeding behavior, consuming a wide variety of prey items, which contributes to this species' high isotopic niche overlap with different salmon species.

In terms of spatial distribution, there was a positive correlation between pink salmon and both coho and chum salmon, but a negative correlation between pink and sockeye salmon (Figure 5G). Previous studies in the North Pacific have pointed to the effect of competitive interactions between pink salmon and other Pacific salmon (Ruggerone et al. 2003; Ruggerone and Connors 2015; Ruggerone and Nielsen 2004; Tadokoro et al. 1996). For instance, there is evidence that the annual growth of sockeye salmon is reduced in years of high abundance of pink salmon (Ruggerone et al. 2003; Ruggerone and Nielsen 2004). Although catches were low for pink salmon overall and especially in the NW‐GoA, this species was detected in the region using environmental DNA (Deeg et al. 2023). Yet, pink salmon and sockeye salmon were not detected in the same oceanographic stations (Deeg et al. 2023). If pink salmon have a negative impact on sockeye salmon populations, it would not be surprising that sockeye salmon avoided pink salmon, as indicated by the negative correlation in spatial distribution found here, at least during winter months when resources are more limited.

Low isotopic niche overlap was detected between pink salmon and non‐salmonid species in the SE‐GoA, except for the squid *O. borealijaponica* and the ctenophore *H. cucumis* (Appendix S3), and there was a positive correlation between the spatial distribution of pink salmon and the squid *O. borealijaponica*. This differs from the negative spatial correlation observed between sockeye salmon and both the squid *O. borealijaponica* and pink salmon. On the other hand, pink salmon were mostly caught in day trawls, while the squid *O. borealijaponica* was mainly caught at night (Appendix S4), which could suggest some degree of spatial segregation due to diel vertical migration. It is important to note, however, that just as with chum salmon, diel vertical migration may provide short‐term spatial segregation but does not necessarily mean that there is less potential for competition since these species may still be eating the same prey. While the generalist feeding behavior of pink salmon might allow this species to coexist despite competition with other meso‐predators that have similar ecological roles, other factors may play an important role, such as pink salmon being the dominant competitor species (Ruggerone and Nielsen 2004). Understanding how different scenarios of competitive interactions, prey abundance, and environmental conditions affect a species' ability to thrive is key to assessing the impacts of increasing temperatures on the North Pacific food web dynamics.

Lastly, our data showed significantly high biomasses of jellyfish (scyphomedusae) in the NW‐GoA, along with high biomasses of sockeye and chum salmon. In contrast, in the SE‐GoA there were high biomasses of the squid *O. borealijaponica*, the myctophid *T. crenularis* , coho, and chum salmon (Figure 6). Isotopic niche overlap was high, especially between salmon and myctophids and squids in the NW‐GoA, but not with jellyfish (Figure 4), despite this group's high biomass in this region. That said, the indirect effect of jellyfish presence on the feeding success of North Pacific meso‐predators should not be overlooked. While the NW‐GoA had the highest biomasses of jellyfish, the SE‐GoA had very low jellyfish biomass and much greater biomasses of zooplankton, myctophids, and squids. Because jellyfish consume zooplankton (Schneider and Behrends 1998; Suchman et al. 2008), they may have had an indirect effect on the meso‐predator species in the NW‐GoA by depleting zooplankton stocks. The impact of jellyfish on the trophic pathways that sustain other meso‐predators has been demonstrated in the northeast Pacific, where the production of coho and Chinook salmon were negatively affected by high biomasses of the scyphozoan jellyfish *Chrysaora fuscescens* (Ruzicka, Daly, and Brodeur 2016). In contrast, large macrozooplankton such as adult *Euphausia pacifica* might be able to avoid predation by jellyfish in the NW‐GoA, with the early larval stages being more vulnerable to predation (Suchman et al. 2008).

Typically, studies exploring interspecific competition consider the interactions between closely related species (Miller, Wilder, and Wilson 2015; Ogloff et al. 2019; Ruggerone et al. 2003; Ruggerone and Nielsen 2004; Taniguchi and Nakano 2000), as they are more likely to use similar resources. However, competition can also occur between unrelated taxa (Andersen et al. 2007; Brown, Davidson, and Reichman 1979; Brown and Davidson 1977; Eriksson 1979), and such interactions have the potential to significantly impact the populations of the involved species. Previous research has addressed the effects of competition on Pacific salmon population dynamics by focusing exclusively on the interactions with other salmonid species (Ruggerone et al. 2003; Ruggerone and Connors 2015; Ruggerone and Nielsen 2004). This study is the first to identify the potential for interguild competition in winter as an important aspect to consider when addressing the effects of competition on Pacific salmon (but see Ruggerone et al. 2023). Competition occurs when species coexist within the same habitats, exhibit a high degree of dietary overlap, and face limitations in their available food resources. In the western North Pacific, which supports large stocks of chum, pink, and sockeye, no significant competition between and among both salmonids and non‐salmon meso‐predators was detected, likely due to the high prey standing stocks in that region (Naydenko and Khoruzhiy 2014; Naydenko and Somov 2022). Here, we have identified substantial overlap in the use of resources among co‐ocurring salmonid and non‐salmonid meso‐predators in the eastern North Pacific during the winter months. However, the intensity of these interactions appears to be species specific and spatially variable. Therefore, our hypothesis that there would be a high isotopic niche overlap between Pacific salmon and other meso‐predators due to limited food availability during winter was only partially supported by our data. This suggests that resource availability likely varies across the Gulf of Alaska and may be influenced by ocean conditions. Our study demonstrated that the Northeast Pacific Ocean is heterogeneous in terms of oceanographic features, with an apparent link between ocean gradients, prey intake by different species, and the level of interspecific interactions. Given the potential for limited food availability during winter and the uncertain effects of climate‐related changes on the distribution of prey and competitor species, potential competitive interactions should be considered as a significant factor when developing food web models to investigate species trends within the eastern North Pacific Ocean.

## 5 Conclusions and Future Directions

In this study, we combined the analysis of stable isotopes and trends in biomass distribution to identify potential competitive interactions among Northeast Pacific meso‐predators during winter months. Dietary data from the stomachs of Pacific salmon helped to interpret isotopic patterns and species interactions. There is little information available on the diets of fish and cephalopod species in open waters of the eastern North Pacific, because of the challenges in making such observations. Therefore, despite the limitations of the method, the use of stable isotopes provides a valuable avenue for studying the feeding ecology of oceanic meso‐predators. Although stable isotope analysis cannot provide information on the prey species or size consumed, it offers invaluable information on consumer's trophic positions and overlap in the use of resources, helping to improve our understanding of the structure of the North Pacific pelagic food webs.

We recognize that the methods used in this study provide dietary and ecological information over different time scales. Stable isotopes integrate diet over a few weeks to months, whereas stomach content data and spatial distribution trends represent the consumers' diet and habitat used immediately before and during sampling. The implication for these differences is that, within the period of isotopic integration for the tissue analyzed (i.e., a couple of months in muscle tissue), a salmon specimen could have explored an area that encompasses our entire survey region, if they had no regional preference. Thus, the longer‐term spatial correlation between two species could deviate from the trends observed during a single sampling event. Despite these differences in time integration, combining these methods revealed that North Pacific food webs and trophic interactions are dynamic across both temporal (daily) and spatial (horizontal and vertical) scales. For example, the high isotopic niche overlap and similar daily water‐depth patterns (i.e., preference for surface waters at night) observed between sockeye salmon and squids were contrasted by a negative longitudinal spatial correlation. Indeed, sockeye biomass was substantially higher in the NW‐GoA, while that of these squids was higher in the SE‐GoA. Accordingly, while these species may overlap in their ecological niches, spatial segregation might be a mechanism to reduce competition for shared resources. Nevertheless, long‐term monitoring programs would be helpful to better understand trends in spatial distributions and to verify whether they persist.

In the current scenarios of climate change and for management purposes, it is essential to gain a thorough understanding of how inter‐specific interactions such as competition are affected by changes in food availability and environmental conditions. Future research should combine the analysis of stomach contents, bulk‐tissue, and compound‐specific stable isotopes to further resolve the degree of dietary overlap among North Pacific meso‐predators over gradients of ocean conditions. Understanding how patterns persist or vary depending on physical and biological gradients may help to shed light on how different species use the available resources, and how species distributions and interactions depend on prey availability and competitor abundances (e.g., between sockeye salmon and the squid *O. borealijaponica*) during the important winter period in the North Pacific high seas. It is also important to take into account increasing energetic demands during the winter months due to ocean warming, that may amplify resource competition.

Our study identified the potential for competitive interactions among meso‐predator species during winter in the Gulf of Alaska. Quantifying the impact that such competition will have on competing species is challenging, especially without the possibility of controlled experiments. Nevertheless, the information provided here can be used to inform food web models to predict the impacts of interspecific trophic interactions on populations and food web dynamics in the eastern North Pacific during winter.

## Author Contributions

**Genyffer C. Troina:** conceptualization (lead), data curation (lead), formal analysis (lead), investigation (equal), methodology (lead), validation (lead), visualization (lead), writing – original draft (lead), writing – review and editing (equal). **Evgeny A. Pakhomov:** investigation (supporting), methodology (supporting), project administration (supporting), validation (supporting), visualization (equal), writing – review and editing (equal). **Laurie Weitkamp:** investigation (supporting), methodology (supporting), validation (equal), writing – review and editing (equal). **Aleksey Somov:** data curation (supporting), methodology (supporting), validation (equal), writing – review and editing (equal). **Brian P. V. Hunt:** conceptualization (equal), funding acquisition (lead), investigation (equal), methodology (equal), project administration (lead), validation (equal), visualization (equal), writing – review and editing (equal).

## Conflicts of Interest

The authors declare no conflicts of interest.

## Supporting information


**Appendix S1.** Biomass correlation between salmon and non‐salmon species sampled in the Gulf of Alaska during the International Year of the Salmon 2019 expedition.

**Appendix S2.** Spatial comparison of trophic positions for those species sampled in both the northwest (NW‐GoA) and southeast (SE‐GoA) Gulf of Alaska.

**Appendix S3.** Isotopic niche overlap between paired salmon and non‐salmon species in the northwestern (NW‐GoA) and southeastern (SE‐GoA) Gulf of Alaska, estimated using Stable Isotope Bayesian Ellipses in R (SIBER).

**Appendix S4.** Relative biomass caught during day and night trawls in the northwest (NW‐GoA) and southeast (SE‐GoA) Gulf of Alaska relative to the total species biomass caught (%).

**Appendix S5.** Estimates of the total biomass of species in the northwest (NW‐GoA) and southeast (SE‐GoA) Gulf of Alaska.

## Data Availability

The data supporting the findings of this study are available in an open‐access database at the International Year of the Salmon Data Mobilization Portal. The dataset can be accessed at https://doi.org/10.21966/jxrd‐8g95.
