# Supplementary material for: The Hunger Games: Stable Isotopes Indicate Winter Inter‐Guild Competition for Resources by Marine Meso‐Predators in the Sub‐Arctic North Pacific
Source: Ecol Evol. 2024 Nov 26;14(11):e70535. doi: 10.1002/ece3.70535 (PMC11597504; doi:10.1002/ece3.70535)
Supplement: Supplementary file 1 — Appendix S1. Biomass correlation between salmon and non‐salmon species sampled in the Gulf of Alaska during the International Year of the Salmon 2019 expedition. [file ECE3-14-e70535-s002.docx]

**Appendix 1:** Biomass correlation between salmon and non-salmon species sampled in the Gulf of Alaska during the International Year of the Salmon 2019 expedition. A) Correlation matrix based on catches from the night trawls. B) Correlation matrix including catches from all stations (sampled during the day and the night). C) Correlation matrix based on catches from the day trawls. Significant *p*-values (< 0.05) are highlighted in bold.

|  | *O. gorbuscha* | *O. keta* | *O. kisutch* | *O nerka* | *O. tshawytscha* |
| --- | --- | --- | --- | --- | --- |
| A) Correlation matrix based on night trawls | | | | | |
| *Oncorhynchus gorbuscha* | - | 0.04 | 0.15 | -0.22 | -0.08 |
| *Oncorhynchus keta* | 0.04 | - | **0.5** | 0.16 | **0.43** |
| *Oncorhynchus kisutch* | 0.15 | **0.5** | - | -0.32 | -0.09 |
| *Oncorhynchus nerka* | -0.22 | 0.16 | -0.32 | - | 0.05 |
| *Oncorhynchus tshawytscha* | -0.08 | **0.43** | -0.09 | 0.05 | - |
| *Diaphus theta* | -0.06 | -0.02 | -0.27 | 0.08 | -0.12 |
| *Lestidiops ringens* | 0.21 | -0.24 | -0.11 | -0.15 | -0.05 |
| *Lipolagus ochotensis* | 0.3 | 0 | -0.14 | -0.11 | -0.05 |
| *Microstomus pacificus* | 0.05 | -0.34 | -0.05 | -0.04 | -0.1 |
| *Stenobrachius leucosparus* | -0.14 | 0.13 | -0.24 | **0.43** | -0.11 |
| *Symbolophorus californiensis* | **0.7** | 0.02 | 0.12 | -0.22 | -0.07 |
| *Tarletonbeania crenularis* | -0.04 | 0.21 | **0.71** | -0.25 | -0.15 |
| *Squalus acanthias* | 0.1 | 0.14 | 0.3 | -0.22 | -0.07 |
| *Abraliopsis felis* | -0.08 | 0.02 | 0.33 | -0.19 | -0.1 |
| *Gonatopsis borealis* | 0.3 | 0.32 | **0.58** | -0.23 | -0.08 |
| *Gonatopsis borealis (juvenile)* | -0.09 | -0.17 | 0.23 | 0.06 | -0.17 |
| *Chiroteuthis calyx* | 0.19 | -0.05 | -0.17 | -0.1 | -0.09 |
| *Gonatus madokai* | -0.09 | -0.28 | -0.17 | **0.55** | -0.06 |
| *Gonatus onyx* | -0.15 | -0.32 | -0.15 | **0.49** | -0.1 |
| *Gonatus onyx (juvenile)* | -0.08 | -0.24 | -0.11 | 0.06 | -0.05 |
| *Gonatus sp.* | -0.08 | **0.43** | -0.09 | 0.05 | **1** |
| *Onykia robusta* | -0.08 | **0.44** | **0.53** | -0.15 | -0.05 |
| *Onychyoteuthis borealijaponica* | 0.31 | 0.16 | **0.57** | -0.3 | -0.05 |
| *Aequorea sp.* | -0.16 | -0.27 | -0.14 | **0.45** | -0.09 |
| *Calycopsis simulans* | -0.16 | -0.33 | -0.27 | 0.09 | -0.1 |
| *Aurelia labiata* | -0.08 | 0.29 | -0.16 | **0.47** | -0.06 |
| *Chrysaora melanaster* | -0.19 | -0.29 | -0.29 | 0.17 | -0.12 |
| *Phacellophoroa camtschatica* | 0.13 | 0.39 | 0.21 | 0.02 | -0.1 |
| *Hormiphora cucumis* | -0.08 | **0.46** | -0.05 | 0.03 | **1** |
| B) Correlation matrix based on all stations (day and night trawls) | | | | | |
| *Oncorhynchus gorbuscha* | - | **0.27** | 0.08 | -0.14 | -0.06 |
| *Oncorhynchus keta* | **0.27** | - | **0.31** | 0.02 | 0.12 |
| *Oncorhynchus kisutch* | 0.08 | 0.31 | - | -0.10 | -0.03 |
| *Oncorhynchus nerka* | -0.14 | 0.02 | -0.10 | - | 0.05 |
| *Oncorhynchus tshawytscha* | -0.06 | 0.12 | -0.03 | 0.05 | - |
| *Diaphus theta* | -0.08 | -0.02 | -0.08 | 0.24 | -0.06 |
| *Lestidiops ringens* | 0.03 | -0.07 | -0.03 | -0.05 | -0.03 |
| *Lipolagus ochotensis* | 0.05 | -0.01 | -0.06 | -0.01 | -0.03 |
| *Microstomus pacificus* | -0.05 | -0.11 | 0.07 | 0.10 | -0.06 |
| *Stenobrachius leucosparus* | -0.09 | 0.02 | -0.07 | **0.52** | -0.05 |
| *Symbolophorus californiensis* | 0.15 | -0.01 | 0.19 | -0.08 | -0.04 |
| *Tarletonbeania crenularis* | -0.08 | 0.03 | **0.72** | 0.02 | -0.07 |
| *Squalus acanthias* | -0.01 | 0.03 | **0.34** | -0.08 | -0.04 |
| *Abraliopsis felis* | -0.07 | -0.01 | **0.39** | -0.01 | -0.05 |
| *Gonatopsis borealis* | -0.01 | 0.05 | **0.62** | 0.04 | -0.02 |
| *Gonatopsis borealis (juvenile)* | -0.10 | -0.06 | **0.35** | **0.27** | -0.08 |
| *Chiroteuthis calyx* | -0.02 | -0.01 | -0.07 | 0.02 | -0.05 |
| *Gonatus madokai* | -0.10 | -0.07 | -0.12 | 0.20 | -0.06 |
| *Gonatus onyx* | -0.09 | -0.09 | -0.01 | **0.55** | -0.05 |
| *Gonatus onyx (juvenile)* | -0.06 | -0.05 | -0.04 | 0.14 | -0.03 |
| *Gonatus sp.* | -0.05 | 0.11 | -0.01 | 0.12 | **0.76** |
| *Onykia robusta* | -0.05 | 0.11 | **0.52** | -0.05 | -0.03 |
| *Onychyoteuthis borealijaponica* | -0.03 | -0.01 | **0.58** | -0.02 | -0.01 |
| *Aequorea sp.* | -0.15 | -0.17 | -0.10 | **0.32** | -0.10 |
| *Calycopsis simulans* | -0.11 | -0.11 | -0.11 | 0.22 | -0.06 |
| *Aurelia labiata* | -0.03 | 0.12 | -0.11 | **0.36** | -0.07 |
| *Chrysaora melanaster* | -0.14 | -0.06 | -0.13 | **0.30** | -0.07 |
| *Phacellophoroa camtschatica* | 0.17 | 0.02 | 0.01 | -0.08 | -0.08 |
| *Hormiphora cucumis* | -0.05 | 0.11 | 0.03 | 0.11 | **0.75** |
| C) Correlation matrix based on day trawls | | | | | |
| *Oncorhynchus gorbuscha* | - | 0.29 | 0.31 | -0.14 | -0.05 |
| *Oncorhynchus keta* | 0.29 | - | **0.66** | 0.01 | 0.06 |
| *Oncorhynchus kisutch* | 0.31 | **0.66** | - | -0.14 | 0.04 |
| *Oncorhynchus nerka* | -0.14 | 0.01 | -0.14 | - | -0.06 |
| *Oncorhynchus tshawytscha* | -0.05 | 0.06 | 0.04 | -0.06 | - |
| *Anotopterus nikparini* | 0.07 | 0.06 | -0.07 | -0.06 | -0.03 |
| *Gasterosteus aculeatus* | -0.07 | -0.08 | -0.07 | -0.06 | -0.03 |
| *Icichthys lockingtoni* | -0.07 | -0.08 | -0.07 | -0.06 | -0.03 |
| *Microstomus pacificus* | -0.07 | -0.08 | 0.07 | -0.06 | -0.03 |
| *Tarletonbeania crenularis* | -0.11 | -0.06 | -0.04 | 0.12 | -0.05 |
| *Gonatopsis borealis* | -0.07 | -0.07 | 0.25 | -0.05 | -0.03 |
| *Gonatopsis borealis (juvenile)* | -0.07 | -0.05 | -0.07 | 0.13 | -0.03 |
| *Chiroteuthis calyx* | -0.07 | 0.01 | -0.07 | -0.06 | -0.03 |
| *Gonatus madokai* | -0.12 | -0.05 | -0.12 | 0.16 | -0.05 |
| *Gonatus onyx (juvenile)* | -0.07 | 0.13 | -0.07 | **0.59** | -0.03 |
| *Gonatidae sp.* | -0.07 | -0.03 | -0.07 | 0.32 | -0.03 |
| *Taonius borealis* | -0.07 | -0.07 | -0.07 | -0.06 | -0.03 |
| *Onychyoteuthis borealijaponica* | -0.07 | -0.07 | 0.26 | -0.06 | -0.03 |
| *Aequorea sp.* | -0.18 | -0.18 | -0.12 | -0.07 | -0.12 |
| *Calycopsis simulans* | -0.12 | -0.12 | -0.09 | -0.10 | -0.05 |
| *Aurelia labiata* | -0.02 | 0.12 | 0.03 | -0.14 | -0.10 |
| *Chrysaora melanaster* | -0.13 | 0.06 | -0.13 | **0.57** | -0.04 |
| *Phacellophoroa camtschatica* | 0.16 | -0.02 | 0.03 | -0.12 | -0.08 |
| *Hormiphora cucumis* | -0.09 | -0.09 | 0.29 | -0.08 | -0.02 |
