# Supplementary material for: The Hunger Games: Stable Isotopes Indicate Winter Inter‐Guild Competition for Resources by Marine Meso‐Predators in the Sub‐Arctic North Pacific
Source: Ecol Evol. 2024 Nov 26;14(11):e70535. doi: 10.1002/ece3.70535 (PMC11597504; doi:10.1002/ece3.70535)
Supplement: Supplementary file 2 — Appendix S2. Spatial comparison of trophic positions for those species sampled in both the northwest (NW‐GoA) and southeast (SE‐GoA) Gulf of Alaska. [file ECE3-14-e70535-s005.docx]

**Appendix 2:** Spatial comparison of trophic positions for those species sampled in both northwest (NW-GoA) and southeast (SE-GoA) Gulf of Alaska. Posterior mode for each area, Bhattacharyya coefficient overlap, the probability of trophic position being higher in the NW-GoA, and the probability of TP being higher in the SE-GoA.

| **Species** | **Posterior mode – NW-GoA** | **Posterior mode – SE-GoA** | **Overlap** | **Prob > NW-GoA** | **Prob > SE-GoA** |
| --- | --- | --- | --- | --- | --- |
| **Salmon** |  |  |  |  |  |
| *Oncorhynchus keta* (chum) | 3.31 | 3.13 | 0.74 | 0.86 | 0.14 |
| *Oncorhynchus kisutch* (coho) | 4.18 | 3.95 | 0.82 | 0.83 | 0.17 |
| *Oncorhynchus nerka* (sockeye) | 3.6 | 3.42 | 0.84 | 0.78 | 0.22 |
| **Fish** |  |  |  |  |  |
| *Tarletonbeania crenularis* | 3.43 | 3.48 | 0.93 | 0.43 | 0.57 |
| **Squid** |  |  |  |  |  |
| *Gonatopsis borealis* | 2.82 | 2.60 | 0.68 | 0.88 | 0.13 |
| *Gonatus onyx* | 3.61 | 2.86 | 0.40 | 0.99 | 0.01 |
| *Onychyoteuthis borealijaponica* | 3.16 | 3.65 | 0.66 | 0.14 | 0.86 |
| **Jellyfish** |  |  |  |  |  |
| *Aurelia labiata* | 2.25 | 2.44 | 0.81 | 0.21 | 0.79 |
| *Chrysaora melanaster* | 2.12 | 2.05 | 0.81 | 0.27 | 0.73 |
| *Phacellophoroa camtschatica* | 2.32 | 3.22 | 0.52 | 0.04 | 0.96 |
