# Supplementary material for: The Hunger Games: Stable Isotopes Indicate Winter Inter‐Guild Competition for Resources by Marine Meso‐Predators in the Sub‐Arctic North Pacific
Source: Ecol Evol. 2024 Nov 26;14(11):e70535. doi: 10.1002/ece3.70535 (PMC11597504; doi:10.1002/ece3.70535)
Supplement: Supplementary file 3 — Appendix S3. Isotopic niche overlap between paired salmon and non‐salmon species in the northwestern (NW‐GoA) and southeastern (SE‐GoA) Gulf of Alaska, estimated using Stable Isotope Bayesian Ellipses in R (SIBER). [file ECE3-14-e70535-s001.docx]

**Appendix 3:** Isotopic niche overlap between paired salmon and non-salmon species in the northwestern (NW-GoA) and southeastern (SE-GoA) Gulf of Alaska, estimated using Stable Isotope Bayesian Ellipses in R (SIBER).

| **NW-GoA** | | | | |  |
| --- | --- | --- | --- | --- | --- |
|  | *O. gorbuscha* | *O. keta* | *O. kisutch* | *O. nerka^1^* | *O. nerka (L)^2^* |
| *Oncorhynchus keta* (chum) | - | - | 0.18 | 0.21 | 0.46 |
| *Oncorhynchus kisutch* (coho) | - | 0.18 | - | 0.45 | 0.23 |
| *Oncorhynchus nerka* (sockeye) | - | 0.21 | 0.45 | - | 0.36 |
| *Oncorhynchus nerka* (sockeye)(L) | - | 0.46 | 0.23 | 0.36 | - |
| Fish | - | 0.10 | 0.06 | 0.10 | 0.10 |
| *Diaphus theta* | - | 0.20 | 0.06 | 0.00 | 0.05 |
| *Stenobrachius leucosparus* | - | 0.34 | 0.11 | 0.09 | 0.15 |
| *Tarletonbeania crenularis* | - | 0.35 | 0.09 | 0.04 | 0.15 |
| *Abraliopsis felis* | - | 0.27 | 0.15 | 0.09 | 0.18 |
| *Chiroteuthis calyx* | - | 0.00 | 0.01 | 0.00 | 0.00 |
| *Gonatus onyx* | - | 0.21 | 0.10 | 0.05 | 0.08 |
| *Onychyoteuthis borealijaponica* | - | 0.28 | 0.15 | 0.23 | 0.25 |
| Hydromedusae | - | 0.07 | 0.12 | 0.08 | 0.05 |
| **SE-GoA** | | | | |  |
|  | *O. gorbuscha* | *O. keta* | *O. kisutch* | *O. nerka* |  |
| *Oncorhynchus gorbuscha* (pink) | - | 0.21 | 0.30 | 0.29 |  |
| *Oncorhynchus keta* (chum) | 0.21 | - | 0.02 | 0.42 |  |
| *Oncorhynchus kisutch* (coho) | 0.30 | 0.02 | - | 0.06 |  |
| *Oncorhynchus nerka* (sockeye) | 0.29 | 0.42 | 0.06 | - |  |
| Fish | 0.07 | 0.06 | 0.04 | 0.04 |  |
| *Tarletonbeania crenularis* | 0.00 | 0.24 | 0.00 | 0.14 |  |
| Squids_grouped | 0.00 | 0.00 | 0.00 | 0.00 |  |
| *Gonatus onyx* | 0.00 | 0.28 | 0.00 | 0.08 |  |
| *Onychyoteuthis borealijaponica* | 0.20 | 0.32 | 0.03 | 0.51 |  |
| Hydromedusae | 0.11 | 0.14 | 0.02 | 0.08 |  |
| Ctenophore (*H.cucumis*) | 0.20 | 0.15 | 0.06 | 0.14 |  |
| Scyphozoa (*P.camtschatica*) | 0.09 | 0.02 | 0.06 | 0.03 |  |

^1^ *Oncorhynchus nerka* (sockeye salmon) 300 mm to 400 mm size class.

^2^ *Oncorhynchus nerka* (sockeye salmon) (L) size class with specimens larger than 400 mm.
