# Supplementary material for: The Hunger Games: Stable Isotopes Indicate Winter Inter‐Guild Competition for Resources by Marine Meso‐Predators in the Sub‐Arctic North Pacific
Source: Ecol Evol. 2024 Nov 26;14(11):e70535. doi: 10.1002/ece3.70535 (PMC11597504; doi:10.1002/ece3.70535)

**Appendix 4:** Relative biomass caught during day and night trawls in the northwest (NW-GoA) and southeast (SE-GoA) Gulf of Alaska relative to the total species biomass caught (%).


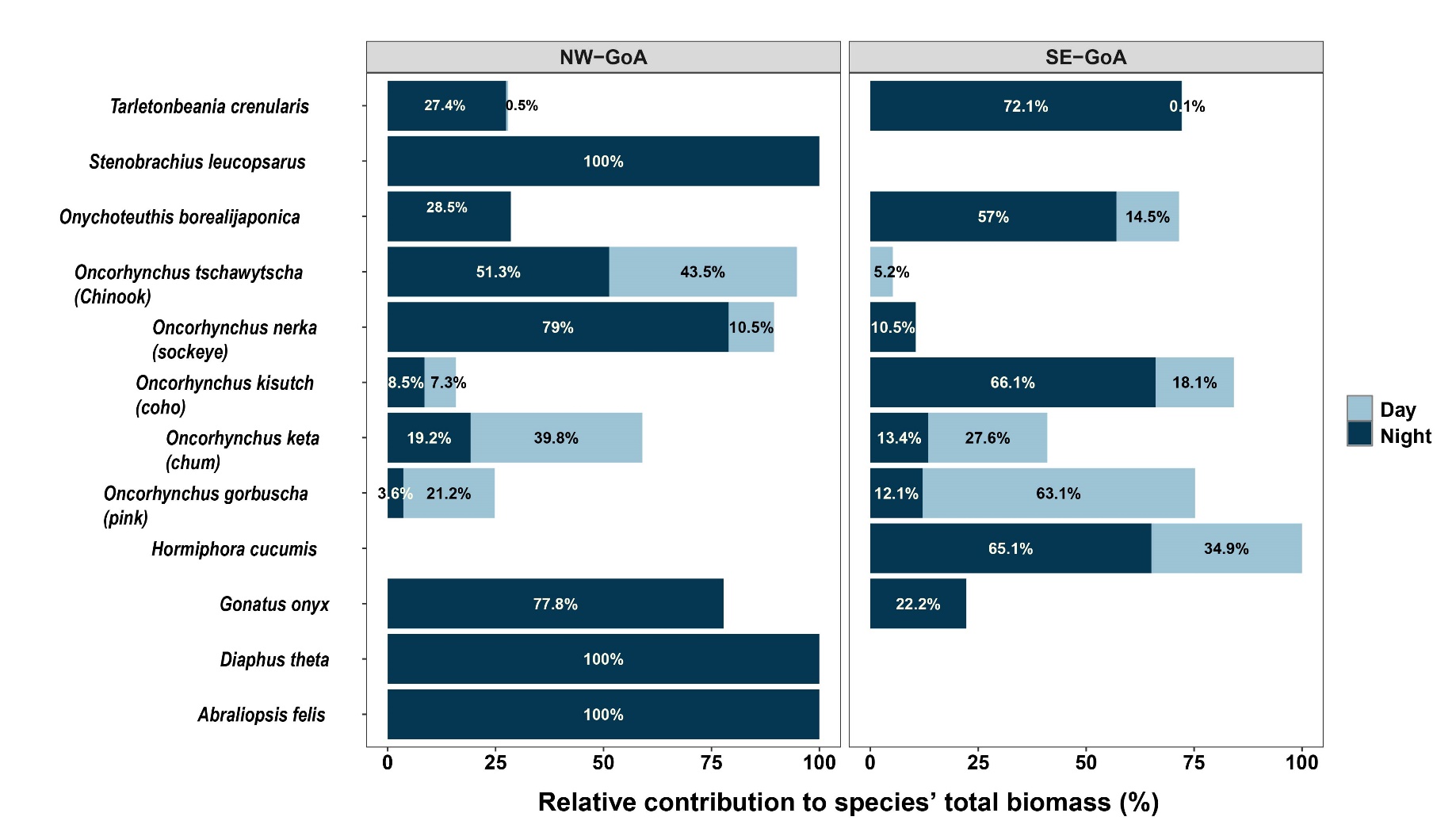

Supplement: Supplementary file 4 — Appendix S4. Relative biomass caught during day and night trawls in the northwest (NW‐GoA) and southeast (SE‐GoA) Gulf of Alaska relative to the total species biomass caught (%). [file ECE3-14-e70535-s003.docx]
