# Supplementary material for: The Hunger Games: Stable Isotopes Indicate Winter Inter‐Guild Competition for Resources by Marine Meso‐Predators in the Sub‐Arctic North Pacific
Source: Ecol Evol. 2024 Nov 26;14(11):e70535. doi: 10.1002/ece3.70535 (PMC11597504; doi:10.1002/ece3.70535)
Supplement: Supplementary file 5 — Appendix S5. Estimates of the total biomass of species in the northwest (NW‐GoA) and southeast (SE‐GoA) Gulf of Alaska. [file ECE3-14-e70535-s004.docx]

**Appendix 5:** Estimates of the total biomass of species in the northwest (NW-GoA) and southeast (SE-GoA) Gulf of Alaska. Biomass was calculated as per area of Voronoi polygon (VP) surveyed in the night trawls and are presented as tons (t) per km^2^. Total Area VP represents the estimated biomass for the entire area surveyed in the NW-GoA (408,445 km^2^) and the SE-GoA (292,166 km^2^).

|  | **Night** | | **Total Area VP** | |
| --- | --- | --- | --- | --- |
| **Species** | **t/km^2^ NW-GoA** | **t/km^2^ SE-GoA** | **t/Total Area – NW-GoA** | **t/Total Area – SE-GoA** |
| **Salmon** |  |  |  |  |
| *Oncorhynchus gorbuscha* (pink) | 0.00041 | 0.00186 | 167.50 | 544.71 |
| *Oncorhynchus keta* (chum) | 0.03651 | 0.03422 | 14,912.55 | 9,998.43 |
| *Oncorhynchus kisutch* (coho) | 0.00604 | 0.06313 | 2,467.24 | 18,445.46 |
| *Oncorhynchus nerka* (sockeye) | 0.05483 | 0.00979 | 22,394.97 | 2,858.85 |
| *Oncorhynchus tschawytscha* (Chinook) | 0.00457 | 0.00000 | 1,866.43 | 0.00 |
| **Other Fish** |  |  |  |  |
| *Diaphus theta* | 0.00271 | 0.00000 | 1,108.51 | 0.00 |
| *Microstomus pacificus* | 0.00000 | 0.00004 | 1.91 | 13.03 |
| *Squalus acanthias* | 0.00000 | 0.00655 | 0.00 | 1,913.21 |
| *Stenobrachius leucosparus* | 0.00070 | 0.00002 | 284.92 | 6.46 |
| *Symbolophorus californiensis* | 0.00003 | 0.00005 | 11.54 | 15.64 |
| *Tarletonbeania crenularis* | 0.00974 | 0.03443 | 3,977.93 | 10,058.36 |
| **Squid** |  |  |  |  |
| *Abraliopsis felis* | 0.00012 | 0.00031 | 49.56 | 90.72 |
| *Gonatopsis borealis* | 0.02760 | 0.05665 | 11,271.06 | 16,551.80 |
| *Gonatopsis borealis (juvenile)* | 0.00810 | 0.01511 | 3,307.07 | 4,414.66 |
| *Chiroteuthis calyx* | 0.00013 | 0.00001 | 51.26 | 2.21 |
| *Gonatus madokai* | 0.00016 | 0.00000 | 64.36 | 0.00 |
| *Gonatus onyx* | 0.00079 | 0.00030 | 322.92 | 88.83 |
| *Gonatus onyx (juvenile)* | 0.00000 | 0.00048 | 0.00 | 139.94 |
| *Gonatus* sp. | 0.00001 | 0.00000 | 3.45 | 0.00 |
| *Onykia robusta* | 0.00000 | 0.00087 | 0.00 | 252.78 |
| *Onychyoteuthis borealijaponica* | 0.01183 | 0.03176 | 4,833.16 | 9,278.08 |
| **Jellyfish** |  |  |  |  |
| *Aequorea sp.* | 0.04024 | 0.00890 | 16,435.27 | 2,599.75 |
| *Calycopsis simulans* | 0.00153 | 0.00052 | 626.82 | 150.70 |
| *Phacellophoroa camtschatica* | 0.01154 | 0.01366 | 4,715.00 | 3,989.58 |
| *Hormiphora cucumis* | 0.02399 | 0.00354 | 9,797.99 | 1,033.44 |
